# Elucidation of Critical Catalyst Layer Phenomena toward High Production Rates for the Electrochemical Conversion of CO to Ethylene

**DOI:** 10.1021/acsami.3c11743

**Published:** 2024-01-08

**Authors:** Danielle Henckel, Prantik Saha, Fry Intia, Audrey K. Taylor, Carlos Baez-Cotto, Leiming Hu, Maarten Schellekens, Hunter Simonson, Elisa M. Miller, Sumit Verma, Scott Mauger, Wilson A. Smith, K. C. Neyerlin

**Affiliations:** † National Renewable Energy Laboratory, 15013 Denver W Parkway, Golden, Colorado 80401-3393, United States; ‡ Department of Chemical and Biological Engineering and Renewable and Sustainable Energy Institute RASEI, University of Colorado Boulder, Boulder, Colorado 80303, United States; § Shell Global Solutions International, B.V., 1031 HW Grasweg 31, Poort 3, Amsterdam 1030 BN, Netherlands; ∥ Shell International Exploration & Production Inc., 3333 Highway 6 South, Houston, Texas 77082, United States

**Keywords:** electrode fabrication, CO reduction, ionomer coverage, hydroxide transport, membrane electrode assembly

## Abstract

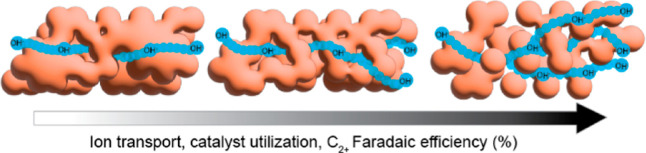

This work utilizes EIS to elucidate the impact of catalyst–ionomer interactions and cathode hydroxide ion transport resistance (*R*_CL_,_OH^–^_) on cell voltage and product selectivity for the electrochemical conversion of CO to ethylene. When using the same Cu catalyst and a Nafion ionomer, varying ink dispersion and electrode deposition methods results in a change of 2 orders of magnitude for *R*_CL_,_OH^–^_ and ca. a 25% change in electrode porosity. Decreasing *R*_CL_,_OH^–^_ results in improved ethylene Faradaic efficiency (FE), up to ∼57%, decrease in hydrogen FE, by ∼36%, and reduction in cell voltage by up to 1 V at 700 mA/cm^2^. Through the optimization of electrode fabrication conditions, we achieve a maximum of 48% ethylene with >90% FE for non-hydrogen products in a 25 cm^2^ membrane electrode assembly at 700 mA/cm^2^ and <3 V. Additionally, the implications of optimizing *R*_CL_,_OH^–^_ is translated to other material requirements, such as anode porosity. We find that the best performing electrodes use ink dispersion and deposition techniques that project well into roll-to-roll processes, demonstrating the scalability of the optimized process.

## Introduction

As renewable electricity continues to become more cost-effective, an abundance of intermittent, low-cost electricity opens the opportunity for electrochemical reactions to provide cost-effective alternatives to current chemical processes. For example, the Fischer–Tropsch reaction, where CO and H_2_ at high temperatures and pressures produce liquid hydrocarbons, could be replaced with electrochemical processes powered by renewable energy.^1^ Electrochemical CO reduction (COR) and CO_2_ reduction (CO_2_R), occurring at lower temperatures (30–80 °C) and pressures (1 atm), can produce C_2+_ products such as ethylene, acetate, ethanol, and n-propanol. While these products can be formed in CO_2_R or COR, COR can improve carbon utilization by avoiding carbonate formation and could be incorporated in tandem reactors (where CO_2_R produces CO and COR produces C_2+_ products).^2−5^ Among the various products resulting from COR, ethylene is particularly of interest with the largest market share of the candidate products aside from n-propanol.^6^ Providing an alternative to industrial reactions such as the Fischer–Tropsch reaction will require large-scale electrochemical devices approaching 1000 cm^2^, significantly beyond what is often observed today in literature.^7^

**Figure 1:**
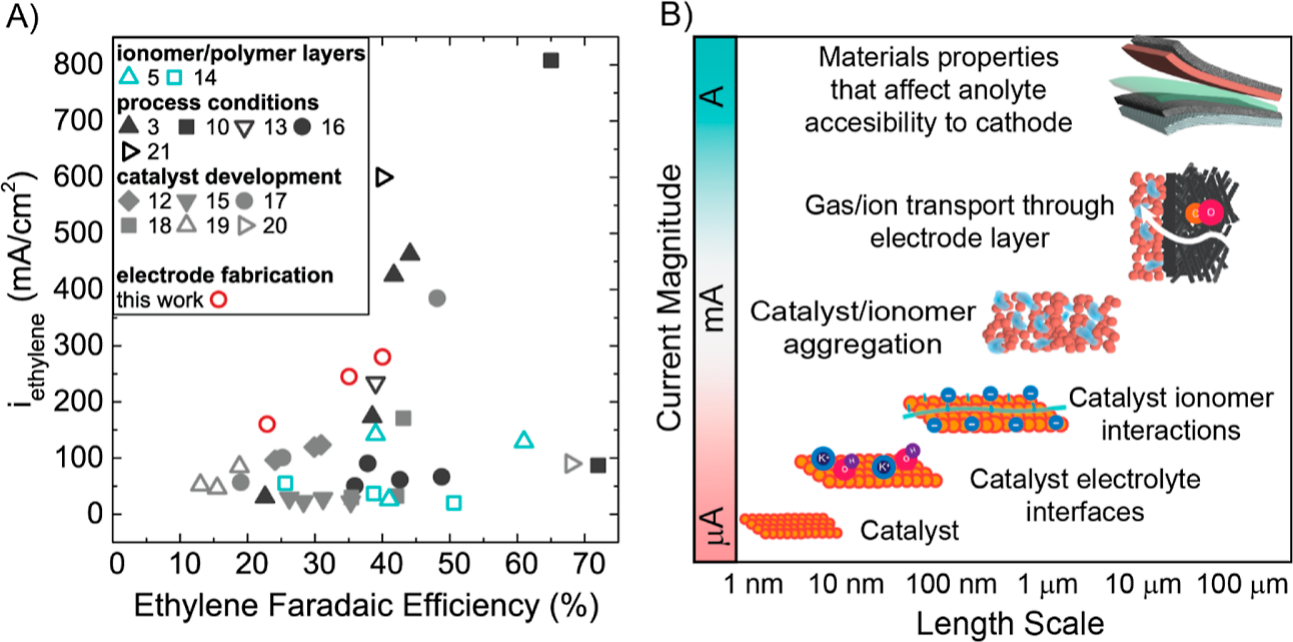
(A) Ethylene FE vs *i*_ethylene_ (mA/cm^2^) from COR publications organized according to focus on process conditions,^3,10,14,17,22^ ionomer polymer layers,^5,15^ or catalyst development^13,16,18−21^ and this work focused on electrode fabrication. The closed symbols indicate a flow cell configuration, and open symbols indicate an MEA configuration. The data from this work are at a current density of 700 mA/cm^2^ and were fabricated with different catalyst deposition methods. (B) Schematic demonstrating factors (interfaces, catalyst layer structure) that affect partial current densities of electrochemical devices and their associated length scales.

Historically, the most common electrochemical device for CO reduction have been H-cells, where the electrode is immersed in an electrolyte saturated in CO. However, these systems achieve only modest current densities (∼10–100 mA/cm^2^) due to low CO solubility and corresponding high mass transport of CO.^8^ Such studies, while allowing relative catalyst improvements, have achieved a maximum of 38 mA/cm^2^ ethylene partial current density.^9^ More recently, reactors utilizing gas diffusion electrodes as catalyst supports and flowing liquid electrolytes have shown increased partial current densities through the introduction of a triple-phase boundary where the gaseous CO and liquid electrolyte meet at the solid interface of the catalyst on the electrode. These systems, utilizing a liquid electrolyte, have shown increased partial current densities to ethylene (*i*_ethylene_) currently as high as 808 mA/cm^2^ (Figure 1A).^10^ Although these systems have desirable partial current densities, the high Ohmic drop across the electrolyte layer drastically decreases the overall energy efficiency of the cell. In order to be cost competitive, electrochemical systems need to operate at >200 mA/cm^2^ with cell voltages less than 4 V.^11^ To that end, membrane electrode assemblies (MEAs), in which electrodes are pressed directly against polymer electrolyte membranes (similar to fuel cells), have been targeted by the research community. For CO/CO_2_ electrolyzers, anion exchange membranes (AEM), such as Aemion+,^12^ are utilized instead to promote a more alkaline environment at the cathode. However, many studies are still being performed in electrochemical cells having an active area of 5 cm^2^ or less, where the focus has been on screening different catalysts/conditions for CO_2_R/COR.^3,5,10,13−21^

To increase the physical scale of CO electrolyzers, it will be necessary to fabricate electrodes with high-throughput scalable processing techniques, capable of producing electrodes at rates approaching 50,000 m^2^/yr to reduce both stack and balance of plant (BOP) costs.^7^ To accelerate the technology readiness level for electrochemical CO conversion, we have studied the transition from lab level electrode production to scalable ink processing and electrode coating methods and the impact on the CO Faradaic efficiency (FE) toward ethylene and an undesirable hydrogen byproduct. Furthermore, we aim to examine this process translation impact in 25 cm^2^ cells at high current densities (700 mA/cm^2^), which are physical and energetic scales closer to industrial applications. In this study, we examine various ink preparation methods including ink mixing (sonication and ball milling) and ionomer content (0–10% Nafion) as well as catalyst deposition methods (hand painting, ultrasonic spray coating, and Mayer rod coating) to understand their impact on electrode level properties that subsequently dictate device level selectivity. The use of commercially available components (Cu nanoparticles and Nafion) was used to ensure the availability of material components in large quantities.

Currently, lab-level, state-of-the-art electrodes for CO/CO_2_R are made using electrodeposition, sputtering, hand painting, or spray coating (aerosol or ultrasonic). While such techniques are useful for tailoring electrode interfaces for devices on the order of 10 s of cm^2^, they will not translate to roll-to-roll (R2R) production rates on the order of 100 s of m^2^ per minute. Contrary to those methods, the Mayer rod coating method utilized here is an intermediate step to the R2R coating of electrode materials methods (such as slot-die, gravure, and knife) as the ink formulation, viscosity, and drying rates are similar to the R2R coating processes. It is relevant to note that the effects of different electrode preparation techniques on the electrode structure and performance have previously been examined in fuel cell literature, with parameters such as drying rate and ink solvent ratios affecting ionomer distribution within the catalyst layer^23^ and electrode mass transport, respectively.^24,25^

In an effort to visualize the progress of the field and showcase the opportunity to improve key performance metrics relevant to increasing the physical and energetic scale of CO electrolysis toward industrial levels utilizing scalable processes, Figure 1A plots the ethylene FE for COR versus ethylene partial current density (*i*_ethylene_ (mA/cm^2^)) obtained across COR publications. The data are sorted according to the methodology used to increase performance: catalyst development (morphology, surface facets), ionomer/polymer overlayers, process conditions, and electrode fabrication methods (this work). While catalyst development primarily affects FE, process conditions (CO flow rates, temperature, electrolyte composition) and ionomer overlayers, in addition to reactor architectures, have increased state-of-the-art *i*_ethylene_. Some techniques may increase ethylene FE, but these effects may not persist at high current densities, thus keeping the performance at lower *i*_ethylene_ values. As we will discuss in this article, the electrode fabrication method has the ability to increase ethylene FE and these effects continue to high current densities, thus also increasing *i*_ethylene_. Correspondingly, Figure 1B shows the compounding factors that affect partial current densities of electrochemical devices and their associated length scales. On the μA scale, while the reaction is under kinetic control, catalyst development, electrolyte and ionomer interfaces dominate performance, as has been noted in recent studies and reviews.^26−29^ However, to achieve higher current densities on the micrometer scale where the reactions will be diffusion-controlled, catalyst/ionomer interactions, catalyst aggregation, and mass and ionic transport through the catalyst layer need to be addressed, all while being achieved through scalable electrode fabrication processes.

In a typical MEA system (without a flowing electrolyte), the ionic conductivity is controlled by the ionomer network in the electrode. In this reactor architecture, a continuous ionomer network is necessary for complete catalyst utilization, along with a high exchange current density and low gas transport resistance, as was demonstrated for fuel cell electrodes previously.^30^ In contrast, when the electrode is in contact with an electrolyte, as in many COR and CO_2_R configurations, ionic accessibility to catalyst sites can be dictated by the electrolyte and the ionomer’s role is not as vital.^31^ While this is true for the abundance of configurations that employ a flowing catholyte, we recently demonstrated that even for pure gas phase cathodes, anolyte crossover dictates ionic accessibility of the cathode catalyst.^32^ Here, we extend that prior diagnostic development effort and understanding to examine the impact of catalyst ink dispersion and deposition techniques on electrode level OH^–^ conduction and catalyst/ionomer interactions in pressed MEA systems. Using a combination of techniques, we describe fundamental characteristics that COR electrodes should possess to promote catalyst utilization and increase product selectivity for MEA style COR devices. The focus of this work is to augment the breakthroughs in catalyst and ionomer development and provide a more direct pathway for scalable component integration.

## Results and Discussion

### Catalyst Ink Processing and Deposition Conditions

**Figure 2:**
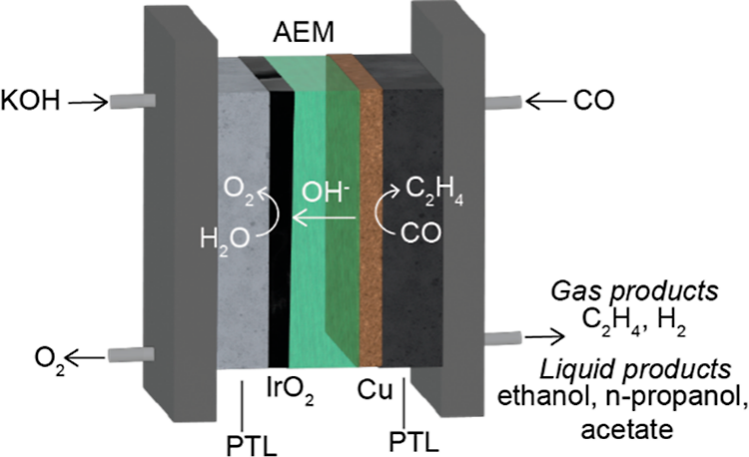
Schematic of the 25 cm^2^ COR cell used in this work, cathode PTL on the right (black) with the Cu catalyst layer atop (brown) separated by the AEM in green from the anode PTL (gray) with an IrO_2_ catalyst layer (black). The anode has a flowing 1 M KOH electrolyte and performs the oxygen evolution reaction (OER). The cathode inlet is humified CO gas, and the COR forms gas (ethylene) and liquid (ethanol, *n*-propanol, and acetate) products, in addition to a hydrogen byproduct.

In order to test the effects of various electrode and ink preparations, we first compared the performance of electrodes made with different ink mixing processes. Two ink mixing techniques, sonication and ball milling (see the Supporting Information for details), were selected due to their ubiquitous appearance in lab-scale and mass production processes.^33^ Ink mixing techniques can impact the desired ink particle size and viscosity, which can then influence the resulting electrode morphology and properties, such as electrochemical surface area, ionic conductivity within the catalyst layer, and electrode mass transport.^25,34−36^ While investigations into ink mixing effects and the resulting electrode performance have been common in the fuel cell literature using carbon-supported Pt particles, this has not been investigated on unsupported (or carbon-free) catalyst materials. For these studies, a common loading (2.7–3.0 mg/cm^2^) of copper catalyst with an oxide content of <32% CuO (Figures S2 and S3) was used along with a constant ionomer content of 0.06 (ionomer/catalyst by mass) and solvent system [1:1 isopropyl alcohol (IPA) and water]. To isolate the impact of ink mixing, ultrasonic spraying (at a flow rate of 0.5 mL/min, denoted as slow spray or SS) was used to fabricate all electrodes. Electrodes were placed in an MEA configuration (see Figure 2) performing COR, where gaseous products were measured by GC and liquid products were measured by HPLC. The full range of FEs can be seen in Figure S5; however, due to product crossover and subsequent oxidation at the anode, it proved difficult to account for all nongaseous products as discussed in prior publications.^14,37^ As an aside to the main focus of this work and to demonstrate the full product distribution, the FE results from an electrode [ball milled ink, rod coated (BM-RC)] run in the H-cell with 1 M KOH are shown in Figure S6. It is relevant to note that recent literature has shown that the product distributions for ethylene and hydrogen do not change significantly from MEA to H-cell testing, though this is likely to depend more on how specific electrochemical properties (e.g., ionic conductivity, gas transport, etc.) vary across testing platforms.^21^

**Figure 3:**
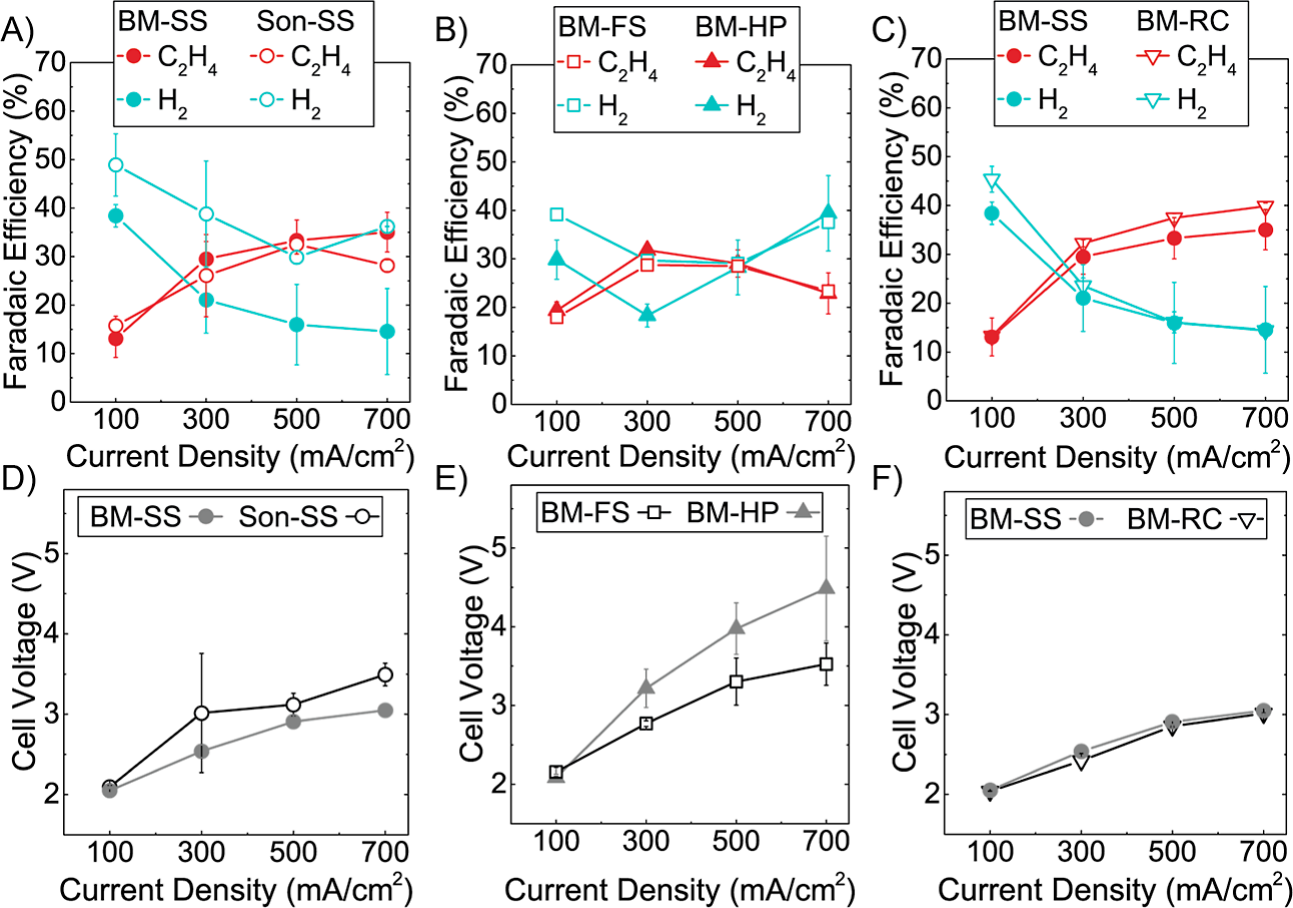
(A–C) FE vs current density (mA/cm^2^) of (A) ball milled (BM) and sonicated (Son) inks deposited by slow spray (0.5 mL/min) ultrasonic spray (SS) (denoted as BM-SS and Son-SS, respectively). (B) BM inks coated by hand painting (HP) and fast spray ultrasonic spray coated at 1.0 mL/min (FS) (denoted as BM-HP and BM-FS, respectively). (C) Mayer rod coated electrodes (RC) and slow flow rate ultrasonic spray-coated inks at 0.5 mL/min (denoted as BM-RC and BM-SS, respectively). (D–F) Cell voltage as a function of current density (mA/cm^2^) for the data in (A–C), respectively. Error bars are the standard deviation from 3 different electrode samples.

Nevertheless, the FE of ethylene and hydrogen from COR in an MEA with sonicated ink, slow flow rate ultrasonic spray (Son-SS) and ball-milled ink, and slow flow rate ultrasonic spray (BM-SS) electrodes are shown in Figure 3A. The ethylene FE of both ink processing techniques is consistently around 30–35%, even at high current densities (700 mA/cm^2^). However, the hydrogen FE is on average 15–20% higher for the Son-SS sample across the entire current density range (the details of which will be discussed in subsequent sections). Due to the lower hydrogen FE, ball milling was used as the ink dispersing technique to examine the impact of various deposition techniques on COR selectivity.

To bridge the gap between more common lab-scale electrode deposition techniques and scalable processes, hand-painting, ultrasonic spray coating (at two different flow rates), and Mayer rod coating were all used to fabricate electrodes, and their performances were compared. It is noteworthy that Mayer rod coating is a commonly used technique for electrode coatings in fuel cell electrode fabrication (decal and direct catalyst coatings); however, it is not common in the COR and CO_2_R literature (see Supporting Information Figure S7 for more details).^23^

Figure 3B shows the FEs for ethylene and hydrogen resulting from hand-painted (BM-HP) and fast flow rate (1.0 mL/min) ultrasonically sprayed (BM-FS) electrodes. Figure 3C displays the same information for rod coated (BM-RC) and slow flow rate (0.5 mL/min) ultrasonic sprayed (BM-SS) electrodes. These techniques were overlaid to show similarities in FE relative to electrode drying behavior. Both the BM-RC and BM-SS create conditions where the IPA and water inks dry more rapidly after deposition on the gas diffusion electrode. The BM-HP and BM-FS electrodes, however, were deposited under conditions where the catalyst ink remains wet after initial deposition, affording opportunities for the ionomer–electrocatalyst interactions and aggregation to change from their ink level behavior. These can be categorized as fast (BM-SS, BM-RC) and slow catalyst drying deposition (BM-HP, BM-FS) techniques, relatively speaking. It is interesting to observe that regardless of the drying time, FE trends across the four techniques remain similar at a 300 mA/cm^2^ total current density with an ∼30–35% ethylene FE and an ∼20–30% hydrogen FE. However, at higher current densities (500–700 mA/cm^2^), the fast-drying techniques (Figure 3C) further increase in ethylene FE, with corresponding decreases in hydrogen FE. In contrast, electrodes made with slow drying techniques show a decrease in ethylene and increase in hydrogen (Figure 2B). Commensurate with the discrepancy in ethylene and hydrogen FE is a disparity in operating voltage, with electrodes made with slow drying techniques yielding significantly higher voltages at 700 mA/cm^2^ (3.5 and 4.5 V for BM-FS and BM-HP, respectively, compared to 3.0 V for BM-RC and BM-SS). Since all MEAs examined above utilize the same anolyte KOH concentration, membrane, and porous transport layer (PTL) set, the added overpotential would indicate that the resulting variations in electrode morphology impact electrochemical properties, namely, OH^–^ conductivity within the cathode as well as the extent of ionomer/electrocatalyst interfacial contact and distribution.

Recent literature has described a correlation between electrode cracking and diminished electrode flooding, and therefore, we have employed both micro-CT (Figure S8) and SEM to characterize such behavior. However, no apparent connection between crack filling or inclusion of the catalyst within the gas diffusion electrode and the ethylene and hydrogen FE is observed in this study.^38^Figures S8–S13 contains cross-sectional and top-down SEM images from the electrodes in Figure 3. Figure S14 shows energy-dispersive X-ray spectroscopy (EDS) mapping of the F content, i.e., the Nafion ionomer. It was observed that the F concentration is higher along the surface cracks in the BM-HP sample, a stark contrast to the other electrodes that had a more uniform distribution. Here, it is worth noting that such cracking features apparent in the deposited electrodes stem from the propagation of features on which they were deposited. The diffusion media material utilized here has cracks in the microporous layer of the same sizes as those observed for the electrodes (Figure S15). Nevertheless, while there is some ionomer aggregation near the cracks for the BM-RC sample, the rest of the ionomer distribution appears on par with the BM-SS sample. While the Son-SS electrode has a more even Nafion distribution, there is more surface ionomer content than the BM-SS. Figure S16 shows the normalized intensities (to Cu) of F from the EDS spectra. The relative Nafion content is as follows—Son-SS > BM-HP > BM-FS > BM-SS ∼ BM-RC where the Son-SS electrode has the most surface ionomer content, and the lowest ionomer content is from fast-drying deposition techniques, BM-RC and BM-SS.

There are several reports where increased mixing time for inks (for our study, sonicated inks are mixed for 30 min and ball milled inks are mixed for 20–24 h) and alternative deposition methods, other than hand painting, show an increased electrode performance. For example, previous work on fuel cell electrodes reported increased mass activity with increased ink mixing, both energetic and temporal.^34^ In addition, ionomer distribution in decal transferred fuel cell electrodes was found to be influenced by the particular manufacturing method, where multiple layer deposition techniques like hand painting creates an ionomer film, whereas single layer deposition blade coating did not.^39^ Jhong et al. has compared the performance of aerosol spray coating and hand painting and found an increase in CO selectivity and CO partial current density from CO_2_R on Ag with the spray coating method.^40^ This increase in performance was attributed to an increase in the coverage of the gas diffusion electrode by the Ag catalyst and a decrease in exposed carbon, but no investigation of the resulting electrodes’ electrochemical properties was performed. For the work presented in this article, the decrease in hydrogen FE between the electrodes with sonicated and ball-milled inks could be related to the resulting ionomer dispersion due to mixing as seen by F-EDS mapping. Although microscopy is a valuable tool, it does not explicitly correlate with the electrochemical properties of the electrodes. For example, although the BM-HP and BM-FS samples appear to have denser electrodes from the SEM top-down images, only electrochemical investigations of ionic conductivity within the electrode can correlate electrode morphology to device level operation, which we discuss in a later section.

Nevertheless, from the data above, the best performing electrodes with the highest ethylene FE at current densities above 300 mA/cm^2^ are from ball-milled inks that are deposited by the Mayer rod or ultrasonic spray at 0.5 mL/min (BM-RC or BM-SS). Despite having similar performances, the rod coating method is preferable, as this method is ∼100x faster with higher throughput than using an ultrasonic spray deposition system.

### Impact of Ionomer Loading on FEs for Ball-Milled, Mayer Rod-Coated Electrodes

The ability of Nafion to promote the formation of C_2+_ products from CO/CO_2_R reactions has been previously reported and attributed to trapping of electrochemically generated OH^–^, thereby increasing the local concentration of OH^–^, which promotes CO dimerization.^2,27,41^ This increase in the concentration of OH^–^ has been shown, in a binary sense, to increase C_2+_ products in CO_2_ reduction.^2,17,27,28,42^ However, the precise trade-off between Nafion content and selectivity for the MEA style CO reduction system has yet to be investigated. As such, we utilized the down selected BM-RC electrodes to examine the impact of ionomer content on COR selectivity and performance.

**Figure 4:**
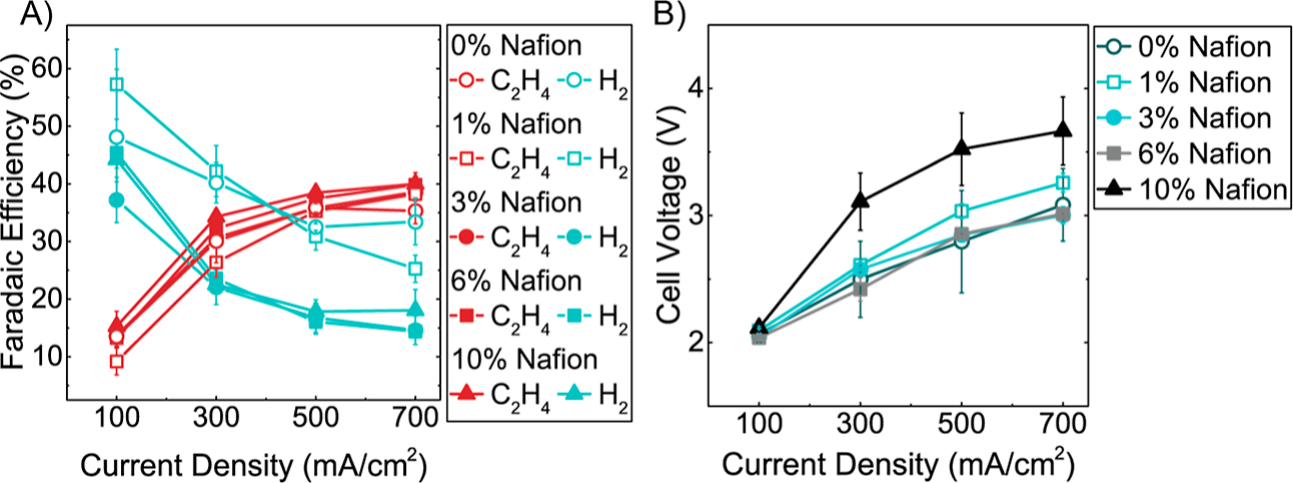
(A) Ethylene and hydrogen FE in BM-RC electrodes as a function of Nafion ionomer loading (0–10% by mass) at 100–700 mA/cm^2^. (B) Cell voltages from data in (A).

Figure 4A shows the ethylene and hydrogen FEs for electrodes fabricated with a range of Nafion weight percentages (0–10%) assessed across a range of applied current densities from 100 to 700 mA/cm^2^. The ethylene FEs are comparable at all Nafion percentages (0–10%) studied here in the range of 35–40%. This outcome is similar to CO_2_R studies performed by Kim et al., where they observed no net change in ethylene production for bare and Nafion1100-coated Cu.^27^ Conversely, hydrogen FEs are significantly impacted by the change in the Nafion content. For electrodes with 1% Nafion and below, hydrogen FE increases to >25% due to the reduced ionomer surface coverage on the Cu electrocatalyst. However, as the Nafion content is increased, there is a significant reduction in the hydrogen FE. For electrodes with >3% Nafion, hydrogen production is suppressed below 15% FE. Due to the promising results of Nafion content >3% and to test our electrodes’ ability to maintain high selectivity at 1 A/cm^2^, we tested the BM-RC 6% electrodes at this current density and obtained 42% ethylene and 14% hydrogen FEs. Again, while the reactant molecule differs from the CO_2_ utilized in the study by Kim et al., the observed impact of Nafion was the same; hydrogen FE was suppressed from ∼20% to 9%, with C_2+_ products increasing from 60 to 70%.^27^

Due to the negative charge on the Nafion side chains, the addition of this polymer to the catalyst layer may enable a more alkaline pH environment due to the Donnan exclusion of OH^–^, effectively trapping the OH^–^ locally produced by COR.^27^ From this study, the extent of this effect appears to be limited to ∼3% Nafion, though variations in ink formulation and ionomer chemistry may change the optimum content. The trends observed with respect to Nafion content utilize one ink mixing and deposition method, but they also shed light on the FE trends from Figure 3A, where the Son-SS electrode had a higher hydrogen FE than the BM-SS electrode. The observed disparity could be due to ineffective mixing of the inks, leading to a relatively higher Nafion content on the surface of the Son-SS electrode (Figure S14), resulting in less Nafion associated with the Cu catalyst throughout the electrode, yielding a fundamental change in both the electrocatalyst microenvironment and the electrochemical properties of the electrode.

### Electrochemical Diagnostics to Elucidate Fundamental Limitations

As mentioned above, differences in electrode performance, and ethylene FE in particular, at higher current densities for the various catalyst ink dispersion and deposition methods cannot be explained by microscopy alone. This is exemplified by the fact that the 0% Nafion electrodes maintain high ethylene FE at high current densities (Figure 3). We have previously published a method to measure electrode-scale properties of a Cu cathode that utilizes EIS.^32^ These properties include, but are not limited to, capacitance (a qualitative measure of catalyst utilization), ionic conductivity (*R*_CL_,_OH^–^_), and catalyst–ionomer interactions (gleaned from the ratio of capacitance at low and high RH). Here, we extend the original technique development to elucidate the impact of ink mixing and deposition on these properties. In an effort to keep the discussion concise, more specific details of the analysis are provided in Supporting Information Figure S17.

When a porous electrode is in contact with an ion-conducting membrane (or liquid electrolyte), ion flux to or from the electrode is maximized at the electrode–membrane interface.^43,44^ Moving away from this interface and toward the diffusion media, the ion flux (OH^–^ flux in this case) decreases, which increases IR drop and thus cell voltage and lowers the reaction rate. Consequently, the rate of electrochemical reactions is also maximized at the electrode–membrane interface and decreases toward the gas side of the diffusion media. When an electrode has very low ionic conductivity, ion flux inside the electrode drops faster and most of the electrode remains unutilized.^30^ Alteration of electrode fabrication conditions (e.g., deposition rate and type, drying rate) leads to a change in the electrode 3D structure (e.g., different particle and agglomerate sizes, different ionomer distributions) that inevitably impacts fundamental properties such as ionic conductivity. Increasing the catalyst utilization will increase the exchange current density^45^ for both COR and HER on Cu. If the electrode has low ionic conductivity, then the COR is confined to the membrane electrode interface, increasing the kinetic overpotential. The competing reaction, HER, only requires water, which should be in abundance and its rate will increase at higher current densities, especially since the reaction can be facilitated on bare carbon,^46^ even in the form of a microporous layer or diffusion media. Thus, electrodes with low ion conductivity (or a high resistance to ion conduction (*R*_CL_,_OH^–^_)) will have lower catalyst utilization and will yield less ethylene.

**Figure 5:**
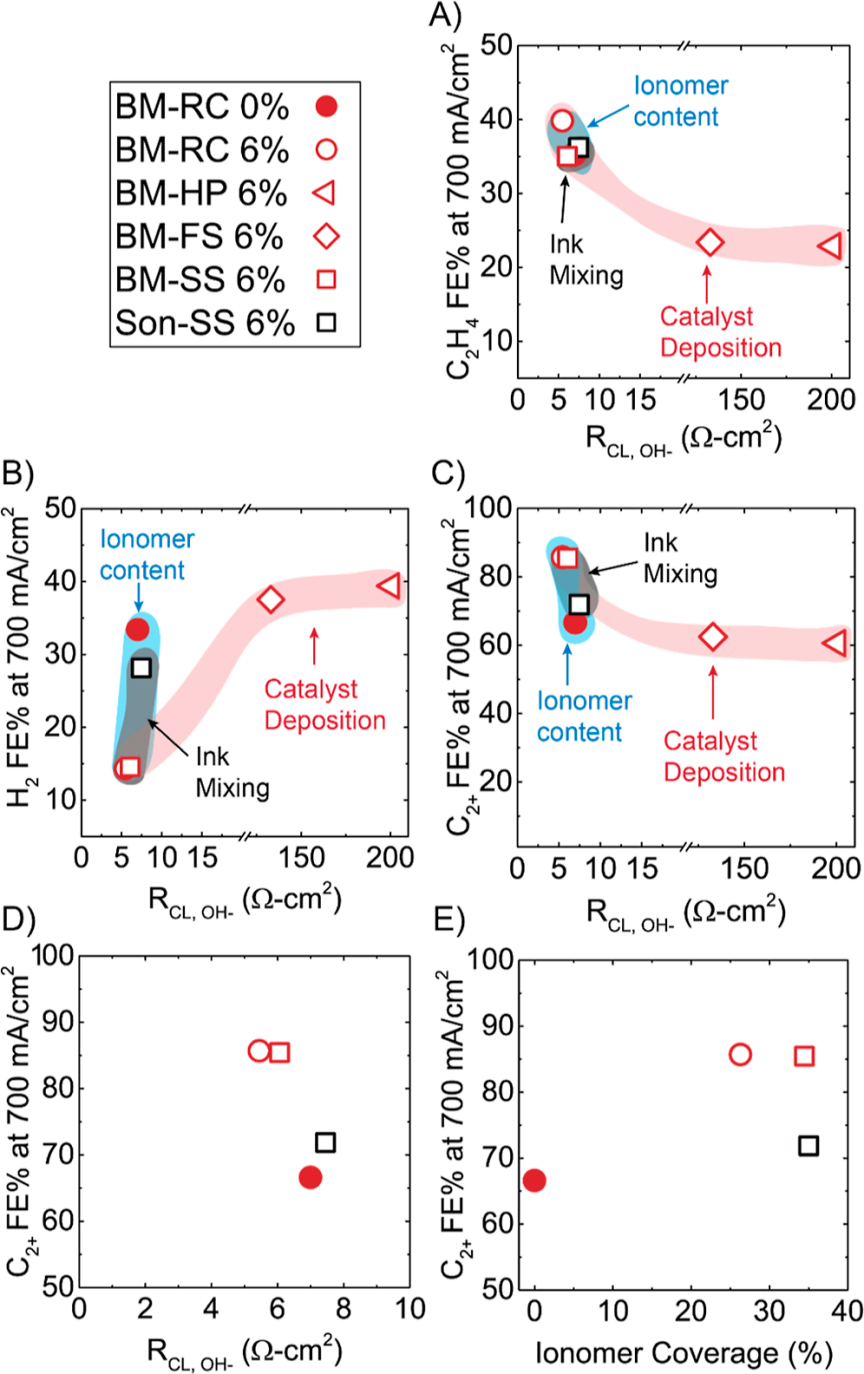
Ion transport resistance (*R*_CL,OH^–^_) vs average (A) ethylene, (B) hydrogen, and (C) C_2+_ FE at 700 mA/cm^2^ (catalyst deposition series—hollow red, ionomer content series—circles, ink mixing—hollow squares). (D) Inset from (C). (E) Ionomer coverage vs C_2+_ FE at 700 mA/cm^2^ for Son-SS 6%, BM-SS 6%, BM-RC 6%, and BM-RC 0%.

Figure 5A–C shows *R*_CL,OH^–^_ (determined from EIS in the presence of 1 M KOH, configuration shown in Figure S17B) plotted vs average FE for ethylene, hydrogen, and C_2+_ products at 700 mA/cm^2^. *R*_CL,OH^–^_ varies widely (nearly 40×) based on the deposition technique. From Figure 5, the samples BM-HP 6% and BM-FS 6% have ionic conductivities (higher *R*_CL,OH^–^_) an order of magnitude lower than those of the electrodes Son-SS, BM-RC 0%, BM-RC 6%, and BM-SS 6%. The result is a much lower catalyst utilization for BM-HP 6% and BM-FS 6%, netting a lower ethylene FE and higher hydrogen FE at 700 mA/cm^2^. *R*_CL,OH^–^_ affects not only the FE but also the cell voltage, which is ∼1 V higher at 700 mA/cm^2^ for these fast-drying electrodes. For the second group of electrodes in 5A, when *R*_CL,OH^–^_ < 7 Ω cm^2^ (Son-SS, BM-RC 0%, BM-RC 6%, and BM-SS 6%), the ethylene FE varies only by 5%. However, when *R*_CL,OH^–^_ > 7 Ω cm^2^, the hydrogen FE varies more considerably, from 12 to 34%, being the highest when no ionomer is present within the electrode.

Nano-CT was utilized to examine the changes in porosity resulting from the fabrication methods (Figure S18). Specifically, the best and worst performing electrodes (BM-RC 6% and BM-HP 6%) were examined to bracket the extremes for *R*_CL,OH^–^_. Figure S18 shows the pore volume distributions and calculated porosities, 56.5 and 30.8%, for the BM-RC 6% and BM-HP 6% electrodes, respectively. The increased porosity for the BM-RC 6% can simultaneously improve gas transport from up to the catalyst sites while also increasing void space near the membrane interface, further promoting back diffusion and transport of the anode electrolyte.

The largest differences in *R*_CL,OH^–^_ can be explained from the slow- versus fast-drying deposition methods, which can be partially attributed here to porosity; however we wanted to investigate differences of the slow-drying electrodes with *R*_CL,OH^–^_ < 7 Ω cm^2^ as it relates to ionomer catalyst interactions. These electrodes (Son-SS, BM-RC 0%, BM-RC 6%, and BM-SS 6%) were made with different ink mixing techniques and ionomer content. To further our analysis, we wanted to probe the catalyst–ionomer interactions of these electrodes. These EIS data were taken without the electrolyte in order to isolate the ionomer interactions. Figure 5E shows the ionomer coverage for these electrodes. The ionomer coverage gives a qualitative estimate of the catalyst–ionomer interfacial area or adsorption interaction. At low RH in this configuration (i.e., no electrolyte), the only available electrochemical interfaces are from the ionomer and not from electrolyte or adsorbed water and this is the only contribution to the double layer capacitance (*C*_DL_).^24,47^ However, at higher RH, due to the water content in the electrode, more ionically conductive pathways are present. Thus, the ratio of *C*_DL_ at 10% RH and *C*_DL_ at 100% RH relates to the catalyst–ionomer interaction. To account for any residual water at 10% RH, we normalized the *C*_DL_ values of the other electrodes to the BM-RC 0% (see Supporting Information Figure S19) as there is no ionomer present in this electrode. Interestingly, as seen in Figure 5E, the ionomer coverage does not appear to be a factor in the FE of the C_2+_ products, with Son-SS 6% and BM-SS 6% having the highest ionomer coverage values. We had originally expected a correlation to ionomer coverage and C_2+_ selectivity due to Nafion’s ability to suppress hydrogen FE. Unexpectedly, the Son-SS 6% electrode has high values for ionomer coverage. Potentially, these improved values for Son-SS 6%, but lack of selectivity, can be explained by the distribution of the ionomer, which lies mostly on the surface.

Although the ionomer content obviously has an effect on the selectivity, the spatial distribution is likely important, and this is not completely encapsulated in the values of ionomer coverage. Regardless, the *R*_CL,OH^–^_ is an excellent indicator for electrode performance (Figure 5D). Overall, the COR FE and performance for a given electrode must be analyzed by considering the sum of the implications from these fundamental properties. The diagnostics utilized here are especially important when studying electrodes to be used in an MEA type system. When interfacing electrodes with electrolytes, such as an H-cell configuration or a flow cell with a catholyte, bulk OH^–^ transport through the electrolyte, and the concentration of OH^–^ in the electrolyte will dominate the effects observed here.

### Anode PTL and the Impact on FE, Performance, and Durability

**Figure 6:**
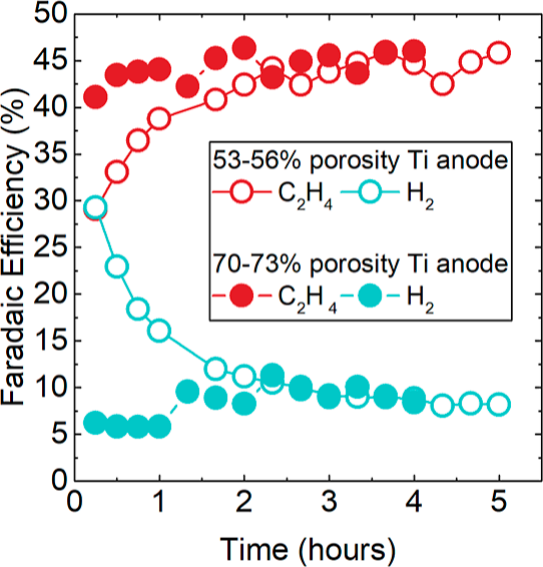
Ethylene and hydrogen FE over time at 500 mA/cm^2^ with BM-RC of 6% using either a higher porosity Ti PTL (70–73%) or a lower porosity Ti PTL (53–56%).

To test the durability of the BM-RC 6% Nafion electrodes, constant current experiments were performed at 500 mA/cm^2^ for 5 and 10 h intervals. The electrochemical cells all used Ti PTLs with an IrO_2_ coating to mitigate anode contributions and isolate cathode degradation. During the initial 10 h experiments, we observed that a break-in time of 2–3 h was required to achieve low hydrogen FE and peak ethylene FE (Figure S20). This contrasts with previous experiments using anodes supported on Toray diffusion media, where we did not observe a needed break-in period for the same performance. Leveraging the understanding revealed both from our prior work, which highlighted the impact of anolyte crossover on catalyst utilization and *R*_CL_,_OH^–^_ inside the cathode^32^ as well as the insight gleaned here, showcasing the relationship between *R*_CL_,_OH^–^_ and ethylene FE, it is apparent that the lower porosity Ti PTLs (53–56%) prevents fast KOH crossover across the membrane to the cathode and slows down the wetting of cathode pores. This decreases the OH^–^ conductivity and catalyst utilization inside the electrode. To facilitate a faster break-in, higher porosity (70–73%) Ti PTLs were examined, and the comparison can be seen in Figure 6. From the outset, the higher porosity Ti PTLs showed significantly lower hydrogen FEs and 10–15% higher ethylene FEs. The impact of the anode PTL porosity dissipates after ca. 3 h, which we attribute to the eventual equilibration of OH^–^ accessibility. This was confirmed through subsequent EIS measurements (Supporting Information for details, Figure S17C). To test our hypothesis of OH^–^ accessibility, we ran in situ EIS at 1 h of operation at 500 mA/cm^2^. After 1 h, the *R*_CL,OH^–^_ of the Cu electrode with high porosity titanium anode is 0.19 Ω cm^2^, whereas the *R*_CL,OH^–^_ in the cell with a low porosity titanium anode is 0.66 Ω cm^2^. Additionally, the capacitance of the cathode paired with the high porosity anode is almost twice (2.3 mF/cm^2^) than that of the cathode paired with the low porosity anode (1.3 mF/cm^2^). This further demonstrates that the crossover from the anolyte is a key parameter in the electrode utilization in an MEA system and that the anode morphology can influence this.^32^

An increase in anolyte concentration (thus anolyte crossover) has been seen in the work by Ozden et al.^5^ As seen in Figure S22, the cell performance from this study increases unilaterally upon increasing the anolyte concentration from 1 to 5 M KOH. This can be attributed to the importance of the electrolyte crossover in changing the [OH^–^] in the catalyst layer, thereby increasing ethylene FE and decreasing ohmic resistance, reducing cell voltage, and increasing energy efficiency.

## Conclusions

**Figure 7:**
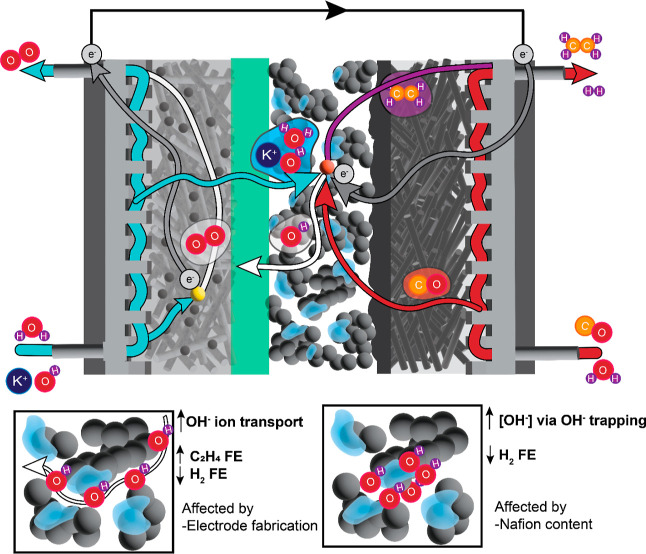
Schematic showing the various processes within the COR cell described here. The cathode reaction, COR, is on the right separated by the AEM (green) from the anode performing the OER. For COR to occur and ethylene to be produced (purple), CO gas (red) must travel through the PTL and the catalyst layer to the reaction site (orange). Additionally, electrons (gray), water (blue), ions OH^–^ (white), and K^+^ (blue) all need to have access to the reaction site.

For COR to take place and outcompete hydrogen production, reactants (CO, H_2_O), ions (OH^–^), and electrons must be present simultaneously to provide an electrochemical active site (Figure 7). Utilizing EIS, we found that *R*_CL,OH^–^_ was a major driver for increased ethylene FE, decreased hydrogen FE, and lower cell voltages at high current densities (>300 mA/cm^2^). This parameter plays a key role in improving catalyst utilization for porous (nonplanar, i.e., sputtered) electrodes. When *R*_CL_,_OH^–^_ is high, the electrode suffers from ion transport deficiencies at higher current densities and, as a result, hydrogen FE increases and C_2+_ FE decreases.

Here, we demonstrated that catalyst ink mixing, catalyst deposition techniques, Nafion content, and anode porosity can all influence COR product selectivity by influencing OH^–^ transport within the catalyst layer. Nafion’s ability to provide OH^–^ trapping, through Donnan exclusion, is nonbinary and depends on the extent to which the ionomer is in contact with the electrocatalyst surface. Ionomer coverage was examined here for a variety of electrodes by using EIS. While significant variations in ionomer coverage on Cu catalysts were observed, trends in C_2+_ FE correlated more with *R*_CL_,_OH^–^_ and electrode porosity, gleaned from nano-CT. Both OH^–^ transport and electrode porosity are impacted by the catalyst deposition method, highlighting the need to focus not only on catalyst optimization and ionomer material sets but also on integration methodology that projects to scalable fabrication for scalable device architectures.

## Supporting Information Available

Experimental details for electrochemical measurements, electrode fabrication, and characterization data including XPS, SEM, micro-CT, and nano-CT (PDF)
