# Supplementary material for: Elucidation of Critical Catalyst Layer Phenomena toward High Production Rates for the Electrochemical Conversion of CO to Ethylene
Source: ACS Appl Mater Interfaces. 2024 Jan 8;16(3):3243–52. doi: 10.1021/acsami.3c11743 (PMC10811620; doi:10.1021/acsami.3c11743)
Supplement: Supplementary file 1 — am3c11743_si_001.pdf [file am3c11743_si_001.pdf]

# Supporting Information

# Elucidation of critical catalyst layer phenomena towards high production rates for the electrochemical conversion of CO to ethylene

*Danielle Henckel[a], Prantik Saha[a], Fry Intia[a], Audrey K. Taylor[a], Carlos Baez-Cotto[a], Leiming Hu[a], Maarten Schellekens[c], Hunter Simonson[a,b], Elisa M. Miller[a], Sumit Verma[d], Scott Mauger[a], Wilson A. Smith[a,b], K.C. Neyerlin[a,*]*

[a]National Renewable Energy Laboratory, 15013 Denver W Parkway, Golden, CO, United States
[b]Department of Chemical and Biological Engineering and Renewable and Sustainable Energy Institute RASEI, University of Colorado Boulder, Boulder, CO, 80303, United States
[c]Shell Global Solutions International, B.V., 1031 HW Grasweg 31, Poort 3, +31 (0) 206 307751, Amsterdam, Netherlands
[d]Shell International Exploration & Production Inc., 3333 Highway 6 South, Houston, TX 77082 USA
*Corresponding author, kenneth.neyerlin@nrel.gov

<u>Source of CO gas:</u>

Prior to results presented here, we initially were not able to observe any ethylene production, observing hydrogen as the only gas product instead. We eventually traced this to carbonyl contamination in the CO source, which was housed in a stainless-steel tank. Stainless steel provides a source of Fe and Ni, which in contact with pressurized CO, leads to the formation of nickel and iron carbonyls.[1] These carbonyls are then likely poisoning the Cu electrodes.[2] This may not have been as influential in earlier studies due to deactivation of the carbonyls in hydroxide electrolytes, which are not in use at the cathode here. We found that the previous CO source contained 169 ppbv Ni and 50 ppmv Fe. These changed to 695 ppbv Ni and 0.2 ppmv Fe after switching to an aluminum tank and an inline CO filter (L-60-MTX, Nanochem, PFR, 60ML) to remove any excess carbonyls. The remainder might be sourced from the stainless-steel

tubing used in the station. Regardless, the current levels do not appear to poison the electrodes under the conditions in this work.

Mayer rod coating:

Mayer rod coating involves the deposition of a viscous ink to a substrate using a rod to draw down the ink in an even layer. Mayer rods are metal rods wrapped with wires of a certain diameter (typically 0.08-2 mm) and the resulting gap spacing controls the wet film thickness and therefore the catalyst loading. As such, Mayer rod coating requires a higher viscosity ink to prevent the ink from freely spreading on the substrate. In contrast, inks used for hand painting and spray coating typically have a lower solids content and a correspondingly low viscosity. To formulate an ink recipe suitable for Mayer rod coating, inks were prepared with 30 wt% Cu, processed using ball milling to disperse the catalyst and ionomer, and then subsequently diluted to 0.6-0.7 wt% for the hand painted and ultrasonic spray coated deposition techniques. These conditions helped to ensure similar particle size distributions were obtained initially prior to deposition.

The Cu cathode catalyst ink was prepared similarly to previously reported work.[3] First, 3.0 g isopropanol (HPLC Plus Grade, 99.9% Sigma Aldrich) and 3.1 g water (18.2 MΩ-cm) were mixed in a 20 mL glass vial and then 0.9 g Nafion® D2020 (Fuel Cell Store) was added. While stirring this mixture with a magnetic stir bar, 3 g of Cu nanoparticles (U.S. Research Nanomaterials, 40 nm or American Elements, <100 nm) were added. After dispersion of the particles in the solvent (1-5 min), the stir bar was removed, and 55 g of 5 mm zirconium oxide beads (Glen Mills) were added. The vial was taped and placed on a Thermo Scientific digital bottle roller at 80 rpm or U.S. Stoneware jar mill roller at 20 speed units (~60 rpm) for 19-24 hours. After milling, the ink was coated at room temperature utilizing a ½” x 16” wire wound

Mayer rod (RD Specialties–25 mil diameter) on a Qualtech automatic film applicator (QPI-AFA6800). The catalyst ink (2-3 mL) was deposited onto a ~5x8 cm Sigracet 39 BB carbon gas diffusion media (Fuel Cell Store) and rod coated at a fixed average speed of 55 mm/s. The catalyst coated gas diffusion media were transferred to an oven and dried at 80 °C. Loading for these electrodes, when Nafion® was used, is consistently 2.7 mg/cm$^2$, but increased to 3.7 mg/cm$^2$ when no Nafion® was used. The loading for Mayer rod coated electrodes were measured from XRF, using a calibration curve of Cu electrodes with known loadings.

Ultrasonic spraying and hand painting:

For the ultrasonic sprayed and hand painted electrodes made with ball-milled inks, the inks were diluted after ball-milling in 1:1 water/IPA solvent mix to 6 mg Cu/ mL solvent. For the ultrasonic sprayed electrodes made with sonicated inks- 108 mg Nafion® D2020 was added to a solvent mixture of 30 g of water and 30 g of IPA, this mixture was shaken and 360 mg Cu was added and horn-sonicated for 2 x 10 seconds at 1 J and then bath sonicated with ice for 30 minutes.

Inks were sprayed with a Sonotek ExactaCoat with Accumist spray nozzle (25 kHz) onto a vacuum hotplate at 80°C. Catalyst inks were loaded into a syringe equipped with a stir bar. The syringe dispensed inks according to a syringe pump and a stir plate was placed below so that the stir bar was constantly stirring at 200-300 rpm. The flow rate of the inks was controlled by a springe pump (Sonotek) at either a flow rate of 1.0 mL/min or 0.5 mL/min (fast flow rate and slow flow rate, respectively).

Hand painted electrodes were painted on a custom made vacuum hotplate heated to 90°C with a paint brush. Loading for hand painted and ultrasonic sprayed electrodes were calculated by mass and were within 2.7-3.0 mg/cm$^2$.

$IrO_2$ and Pt electrode deposition:

$IrO_2$ inks were prepared by mixing 180 mg of 99.98% purity $IrO_2$ powder (Alpha Aesar Premion, 99.99% purity) first with 36 g of 18 MΩ-cm distilled water. This mixture was then horn sonicated for 2 x 10 seconds at 1 J and then bath sonicated with ice for 20 minutes. In a separate 30 mL vial, 270 mg of a 10% perfluorinated anion exchange ionomer (PFAEM)[4] in IPA solution was combined with 24 g of n-propanol (nPA, HPLC Plus Grade, OmniSolv) and this solution was then shaken to combine. After the sonication of the $IrO_2$/water mixture was completed, the PFAEM/nPA mixture was added and the vial shaken. The ink was ultrasonic spray coated (with a flow rate of 1.0 mL/min) onto 4 25 cm$^2$ Toray paper 5% wet-proofed (Fuel Cell Store) or titanium mesh (Fuel Cell Store) placed on a vacuum hotplate set to 80°C. This resulted in an $IrO_2$ loading of ~0.4 mg cm$^2$. For the Pt and $IrO_2$ coated Ti mesh electrodes, the Pt was further coated onto the $IrO_2$ coated Ti mesh electrodes. For this ink, 180 mg of Pt black (Fuel Cell Store) was measured and 36 mg of water was added. After the water addition, 24 g nPA was added to the Pt mixture and finally 180 mg of Nafion® D2020 was added. This ink was sonicated and deposited in the same way as the $IrO_2$ inks.

For the electrochemical impedance measurements, Pt/C electrodes were used. The catalyst ink was prepared by first combining Pt/HSC (Tanaka Kikinzoku Kyogo (TEC10E50E, 46.7 wt% Pt)) with 18.2 MΩ water and then adding Nafion® D2020, and finally nPA. This formulated recipe contained 5 wt. % catalyst, an ionomer-to-carbon weight ratio of 0.2 or 0.9, and a water-to-alcohol weight

ratio of 1.5 (60 wt% $H_2O$). The ink (60 g) was dispersed using the Ultra-Turrax® high-shear rotor-stator with (dispersing element 18G) at 10,000 rpm for 30 min. The vial was then placed on a Fisherbrand™ Digital Bottle Roller at 80 rpm for 1 h to remove any bubbles. Pt/HSC inks were coated at 22 °C utilizing a ½" x 16" wire wound lab rod (RD Specialties–mil diameter = 30) on a Qualtech automatic film applicator (QPI-AFA6800). The catalyst ink (500 µL) was deposited on Sigracet 39 BB (Fuel Cell Store) and rod coated at an average speed of 55 mm/s. These electrodes were transferred to an oven and dried at 80 °C. The average Pt loading was 0.2 mg/$cm^2$.

Oxide content of the catalyst:

Early in this study, we observed inconsistent ionomer loading trends on ethylene FE% across different catalyst batches/suppliers. We found that the varying concentration of CuO, in the catalyst ink explained our results. Figure S1 shows the FE% of ethylene and hydrogen with oxidized Cu (60% CuO via XPS, Figure S1) as a function of ionomer loading. While the effect of Nafion® percentage on hydrogen FE appears to be limited to a Nafion® content of ≤1% on Cu NPs (32% CuO) in Figure 4, this is not true of electrodes made with more CuO content. As seen in Figure S1, the effect of Nafion® extends to ≤6%. The XPS for the electrodes used in the main manuscript can be seen in Figure S2 and S3. While there are reports of oxide-derived copper catalysts increasing $C_{2+}$ FE from CO reduction, we note that the catalyst, ink mixing and catalyst deposition techniques are different than reported here.[5,6]

H-cell operation:

The H-cell results were taken in a custom two compartment cell with 1 M KOH as the catholyte and a 20 mL/min CO flow.

Pre-assembly of membrane electrode assembly:

To prepare for carbon monoxide electrolysis testing, the cathode controlled evaporative mixer was set (Bronkhorst) to 115 °C and the chiller was set to -10 °C. Two 1 L bottles of 1 M KOH were prepared for testing.

Membranes:

For the performance tests, the 50 or 15 μm AF2-HLF8 Ionomr Aemion+® membranes were conditioned through a 1 M KOH 16 hour minimum soak (83% purity Sigma Aldrich KOH pellets), then rinsed in 18 MΩ-cm water preceding cell assembly. All experiments in Figures 3 and 4 were tested with 50 μm AF2-HLF8 Ionomr Aemion+® membranes, except the BM-FS electrodes which were tested with 15 μm AF3-HLF9 Ionomr Aemion+® membranes due to availability. For durability tests, Versogen PiperION® 40 μm membranes were used. These were conditioned similarly to the Ionomr membranes above.

Membrane Electrode Assembly:

For MEA testing, we utilized a custom 25 $cm^2$ cell described previously.[7] To begin the cell assembly, the titanium cathode flow field was evenly sanded with 500 grit sandpaper and distilled water on a leveled block to strip away the titanium oxide layer and lower flow field resistance. Both the thicknesses of the cathode and anode gas diffusion electrodes (GDEs) were measured out. Using these measurements, the ideal gasket thickness was calculated to reach a 20% compression of the electrodes. Then 25 $cm^2$ active area PTFE gaskets with the thickness closest to the ideal were chosen. Using eight bolts and Belleville spring washers, the cell was tightened to 40 in-lbs.

From anode to cathode the cell assembly went as follows: anode end plate, PTFE coated fiberglass separator, gold plated current collector, platinum coated nickel serpentine anode flow

field, anode GDE, PTFE gasket enabling 20% compression, rinsed (18 MΩ-cm water) membrane, PTFE gasket enabling 20% compression for the cathode, cathode GDE, titanium serpentine cathode flow field, gold plated current collector, PTFE coated fiberglass separator and cathode end plate.

Cell Start-up:

For both galvanodynamic and galvanostatic experiments, the running conditions were kept constant. For start-up, the cell was heated to 60 °C at 95% RH with a flow of 1 SLPM of nitrogen through a stainless-steel line set to 70 °C. Care was taken to ensure that 95% cathode gas RH was maintained during the heat-up period. To keep a stable cell temperature at 60 °C, the 1 M KOH anolyte was heated to 55 °C and set to a flow rate of 50 mL $min^{-1}$ (KNF Simdos 10 diaphragm pump). This was all operated at an absolute pressure of 101 kPa controlled by a back pressure regulator (Equilibar). A Gamry 3000 Potentiostat and 30k booster were used for all electrochemical testing. An additional hydration time of 1 hour was necessary for the electrodes with 10% Nafion content, which occurred after cell was brought to temperature, but before the break-in provided below.

Break-in:

Prior to the break-in procedure, 1 SLPM carbon monoxide was provided by a mass flow controller (Alicat MCV-series) and introduced into the cell. The break-in procedure consisted of two galvanodynamic scans from 1 to 500 mA $cm^{-2}$ with the first at 1 mA $cm^{-2}s^{-1}$ and then the second at 5 mA $cm^{-2}s^{-1}$.

Electrochemical testing:

The galvanostatic holds were run at 0.1, 0.3, 0.5, 0.7, and 1 A $cm^{-2}$ for 5 minutes each. During these holds, liquid sampling of the anolyte was taken at 2.5 minutes and gas sampling was taken at 3 minutes. The liquid sampling utilized a 20 mL vial at the end of the anolyte outlet from the cell for 6 seconds and was conducted in the hood to maintain safe practices around carbon monoxide gas. A gas sampling bag was inserted to the gas outlet tubing for 20 seconds. At the end of the cell testing a liquid sample from the cathode condenser was taken.

After all 5 of the galvanostatic holds were conducted, a third galvanodynamic sweep is performed for end of test analysis.

Table S1. Conditioning and testing protocol

| | |
|---|---|
| Conditioning | • Galvanodynamic 1 mA $cm^{-2}s^{-1}$ scan rate, OCV-500 mA/$cm^2$, CO 1 SLPM<br>• Galvanodynamic 5 mA $cm^{-2}s^{-1}$ scan rate, OCV-500 mA/$cm^2$, CO 1 SLPM |
| Galvanostatic hold | • For short performance studies: 100, 300, 500, and 700 mA/$cm^2$<br>• For durability: 500 mA/$cm^2$ for 5-10 hours |

Product Analysis:

Gas analysis was run on the Agilent 990 Micro Gas Chromatography System analyzing quantities of ethylene and hydrogen. Liquid analysis was run on the Agilent 1200 High Performance Liquid Chromatography System with anolyte samples being run without dilution

and cathode condenser samples being run with a dilution to 25 mL with 18 MΩ water. Due to the demonstration from the H-cell with the full product analysis, we calculated the $C_{2+}$ product FE from the membrane electrode assembly (MEA) configuration by subtracting the measured hydrogen FE. This is due to product crossover and oxidation by the anode in the MEA configuration.

Electrochemical impedance spectroscopy (EIS):

As the details of this method are already published, we mention the essential aspects of the novel method in this section.[3] The method is based on a MEA architecture with the following components- a Pt supported on high surface area carbon GDE typically used in proton exchange membrane (PEM) fuel cells, membrane of choice, and cathode GDEs (BM-SS, BM-RC, BM-HP, Son-SS etc.). Hydrogen was flowed through the Pt GDE while $N_2$ was flowed through the Cu GDE. Thus, the Pt GDE was a stable reference/counter electrode while the Cu GDE was used as the working electrode. In this configuration, we control the potential on the working electrode precisely and perform electrochemical impedance spectroscopy (EIS). We ensured capacitive charging at the cathode GDE by $N_2$ flow and minimized the complications arising due to Faradaic reactions through the chosen potential. Then, the EIS data for different cathode GDEs were fitted against the transmission line model of porous electrodes to extract capacitance (a qualitative measure of catalyst utilization) and ionic conductivity inside the electrode.

The different MEA designs were used to analyze- 1) ionomer coverage (Setup A) and 2) ionic conductivity and capacitance inside the cathode GDE (Setup B, C). The experimental procedure is depicted in Figure S17A,B and C. The experimental protocols were the following:

Setup A) 0.3 SLPM of $H_2$ was flowed at the Pt/C counter/reference electrode and $N_2$ was flowed at the Cu working electrode. During the low RH experiment, we kept the $H_2$ gas RH to 40% and the $N_2$ gas RH to 10%. As explained previous work, only the catalyst-ionomer interface is electrochemically active at low RH. During high RH measurements, both the $H_2$ and $N_2$ gas RHs were kept 100%. 100 mV DC + 10 mV AC RMS voltage was applied for the EIS as the charging was capacitive for this potential. The MEA was hydrated for 5-6 hours before the EIS were performed. Nafion® XL membrane was used instead of Nafion® 211 to prevent electrical shorts during dry EIS measurements. The Pt/C GDE had an ionomer-to-carbon ratio of 0.9.

Setup B) The cell was assembled in the order of- Pt/C GDE, AEM, electrolyte channels, a second AEM and finally the Cu GDE. The gas flows ($H_2$ and $N_2$) were lowered to 0.1 SLPM. The electrolyte, 1 M KOH, was flowed in the electrolyte channels at 20 mL/min. The gas flows were reduced to minimize the turbulence caused by the two-phase flows. The same cell voltage as setup A (100 mV DC + 10 mV RMS AC) was used for EIS. The RH at both ends was kept at 95% to maximize the catalyze utilization. At low frequencies, we observed small deviations from ideal EIS spectra caused by the non-steady-state situations arising from the KOH flow inside the GDE. Despite deviations, the trends of the EIS spectra were clear to interpret the data physically. The Pt/C electrode had an ionomer-to-carbon ratio of 0.2 (lower than setup A) because this was an alkaline environment.

Setup C) For the EIS described in the durability section, we used a modified cell set-up (Figure S17C). In this MEA, we used a dual purpose anode- a Ti mesh coated with both Pt and $IrO_2$. During COR operation, 1 M KOH was flow through the anode compartment. Before EIS was taken, the KOH was emptied and 1 SLPM $H_2$ was introduced to provide a stable reference electrode for this data.

Nano-Xray computed tomography (nano-CT):

BM-HP 6% and BM-RC 6% samples were tested with X-ray rouce of 8.0 keV (Zeiss Xradia 800 Ultra) using absorption mode with camera binning 2. The sample was mounted on a stainless-steel pin and rotated from angle -90° to 90° and with an exposure time of 120 s for each image. A total of 901 images were collected during the scan. The filter back projection method was used for the 3D structure reconstruction and has a voxel size of 32 nm. The segmentation and particle size distribution analysis were conducted with custom written code.

Scanning electron microscopy (SEM)

Scanning electron microscopy (SEM) images were collected using the NovaNanoSEM 630 using a secondary electron detection mode, 10 kV, spot size 3, WD 5 mm. EDS mapping performed using a 10 keV energy range with a pixel dwell time 100 µs. The software Aztec Version 6.0 was used for EDS-mapping, with a resolution of 512 by 512 pixels for each map.

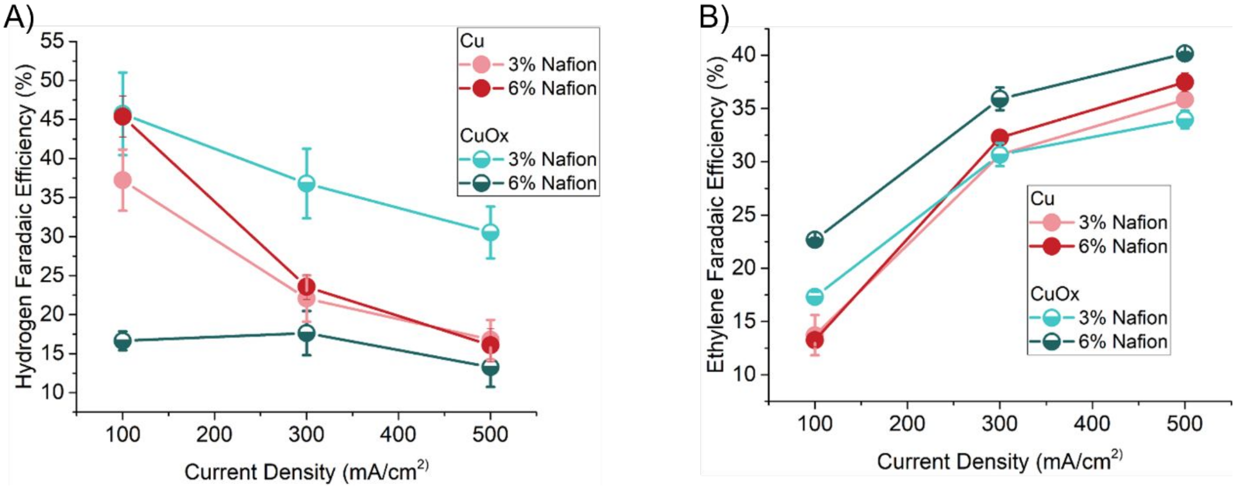


Figure S1. Faradaic efficiencies from CuOx (60% CuO) and Cu (32% CuO) electrodes that were fabricated from ball milled inks and rod -coated with 3 and 6% Nafion® for A) hydrogen B) ethylene

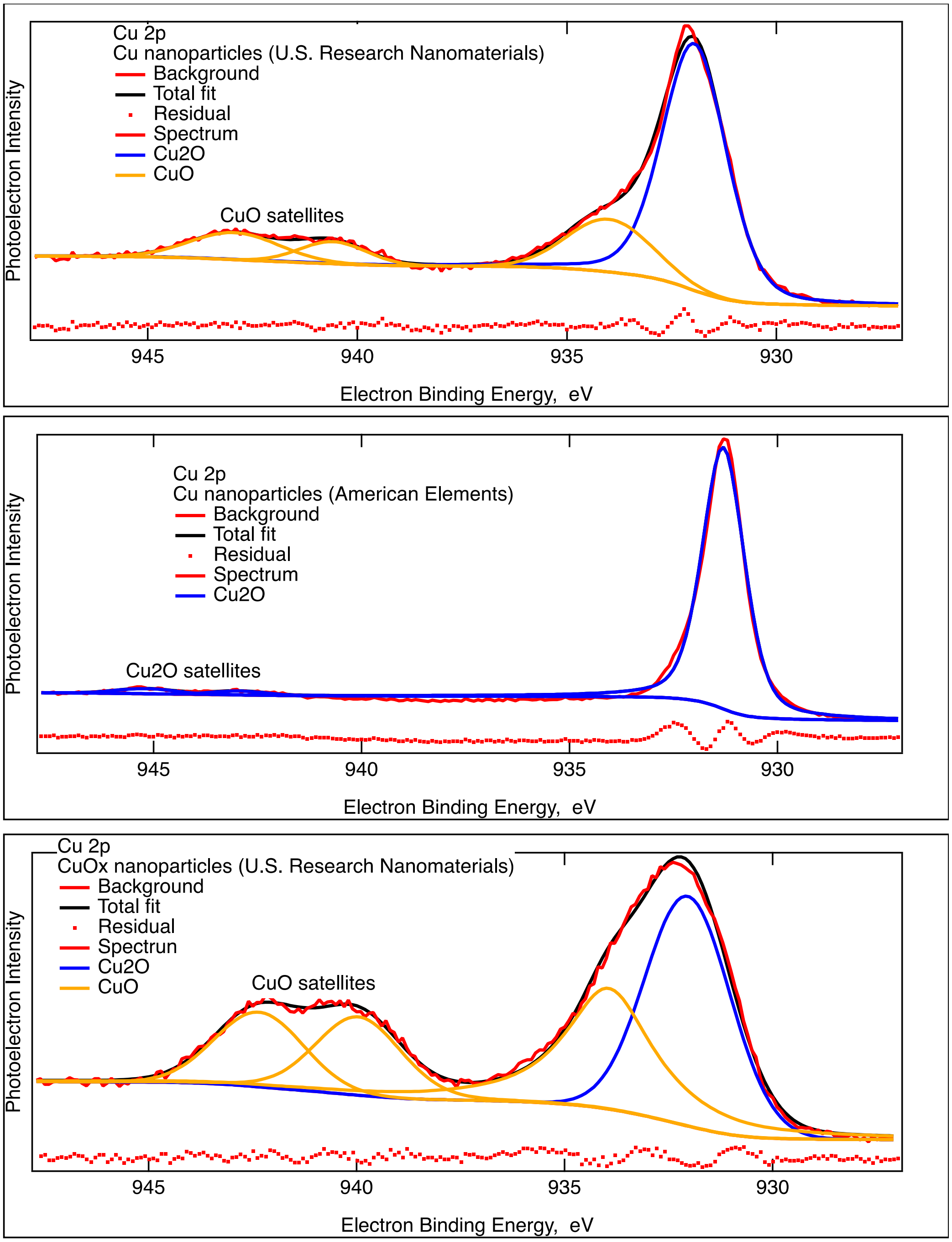


Figure S2: Top: Cu XPS for Cu nanoparticle catalysts from U.S. Research Nanomaterials, 40 nm, Middle: Cu nanoparticle catalysts from American Elements, <100 nm, Bottom: more oxidized Cu nanoparticle catalysts from U.S. Research Nanomaterials, 40 nm from data in Fig. S1, labeled CuOx.

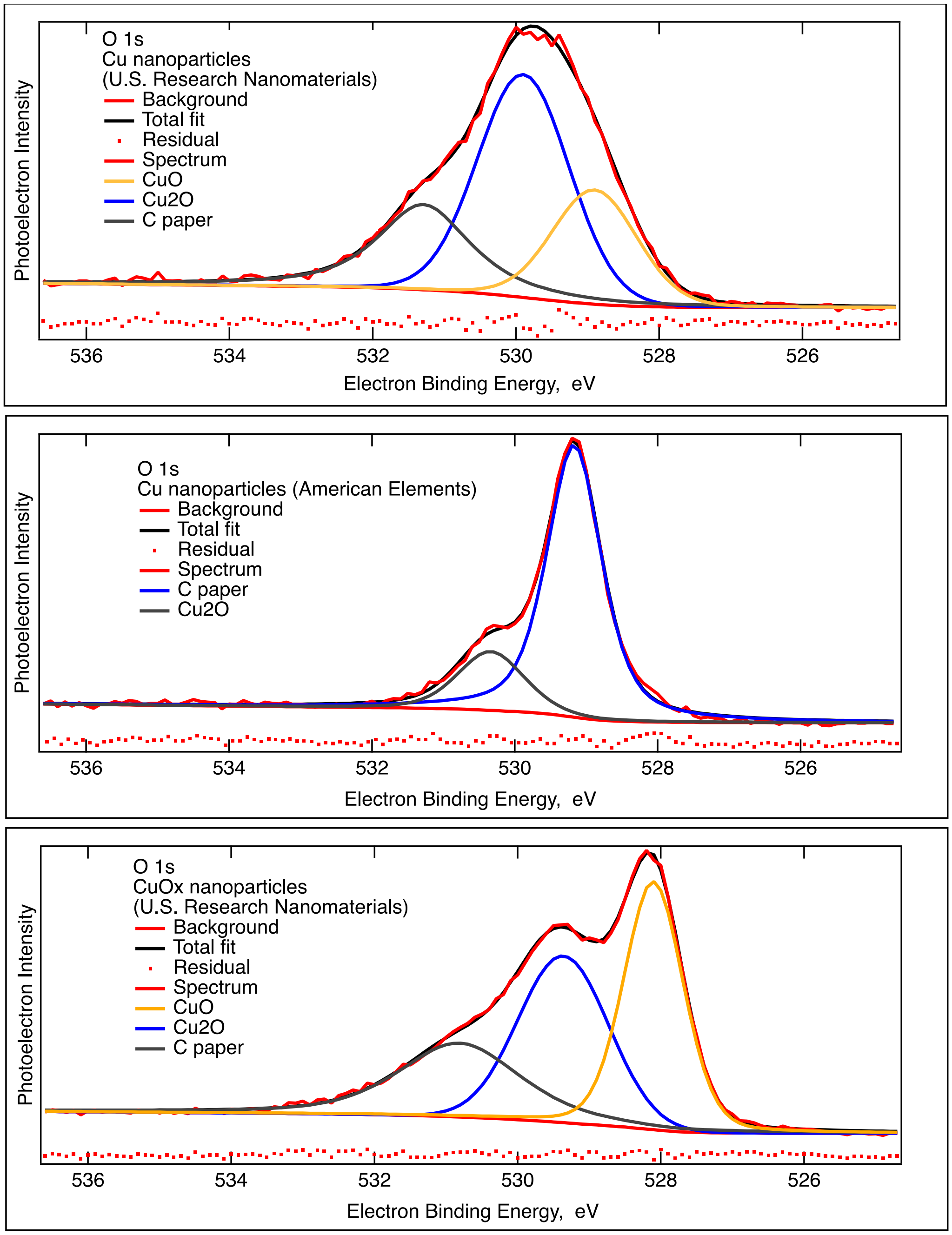


Figure S3: Top: Oxygen XPS for Cu nanoparticle catalysts from U.S. Research Nanomaterials, 40 nm, Middle: Cu nanoparticle catalysts from American Elements, <100 nm, Bottom: more oxidized Cu nanoparticle catalysts from U.S. Research Nanomaterials, 40 nm from data in Fig. S1, labeled CuOx.

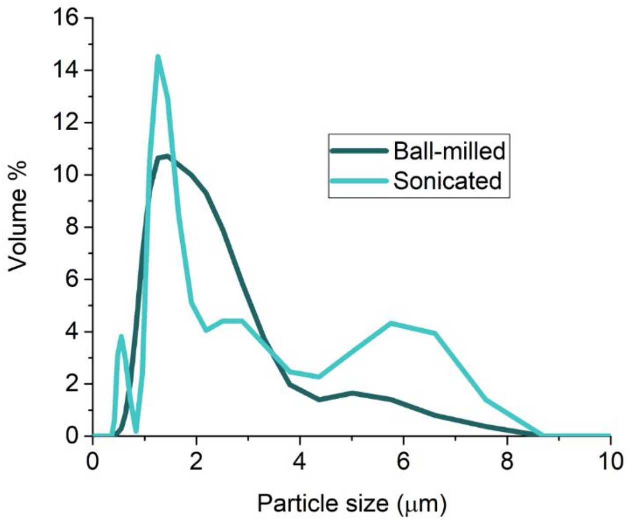


Figure S4. Particle size distributions of ball-milled and sonicated inks (0.5 wt% Cu in 1:1 IPA:water))

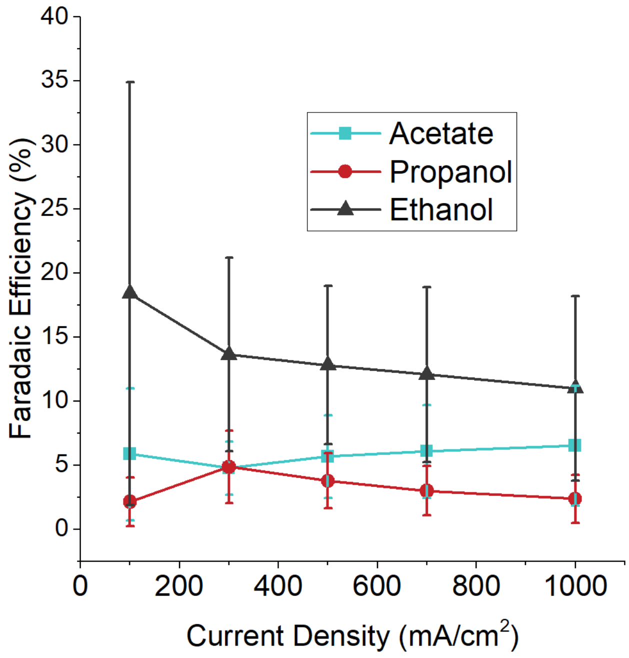


Figure S5. Liquid product distributions analyzed from HPLC from BM-RC 6% (ball-milled, rod-coated, 6% Nafion®) from the MEA configuration with 1 M KOH anolyte.

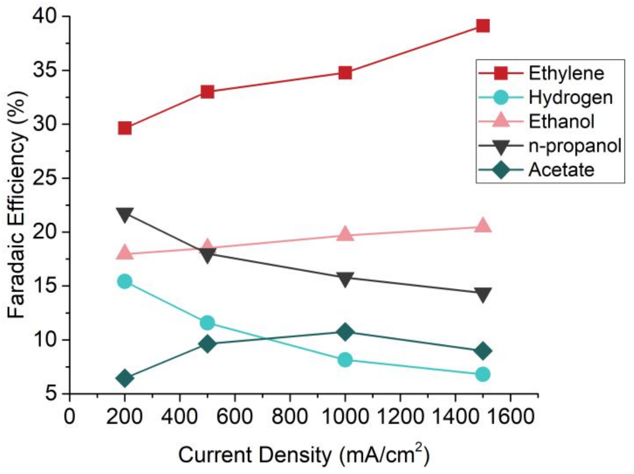


Figure S6. Product distributions from BM-RC 6% (ball-milled, rod-coated, 6% Nafion®) from an H-cell configuration with 1 M KOH catholyte.

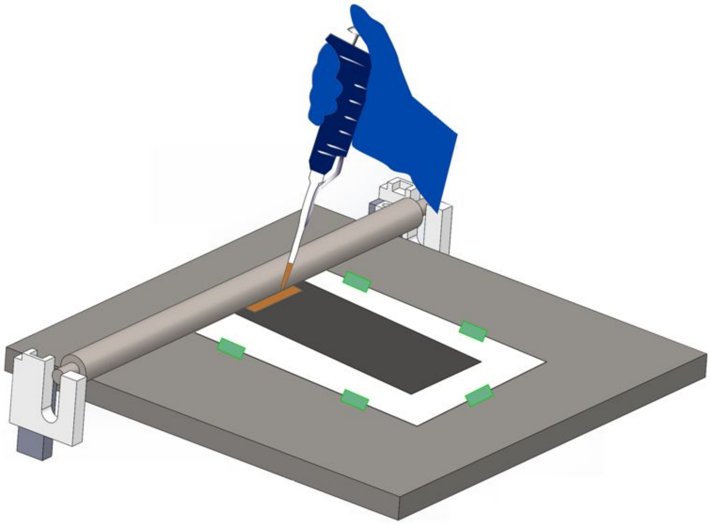

Figure S7. Schematic of Mayer rod coating

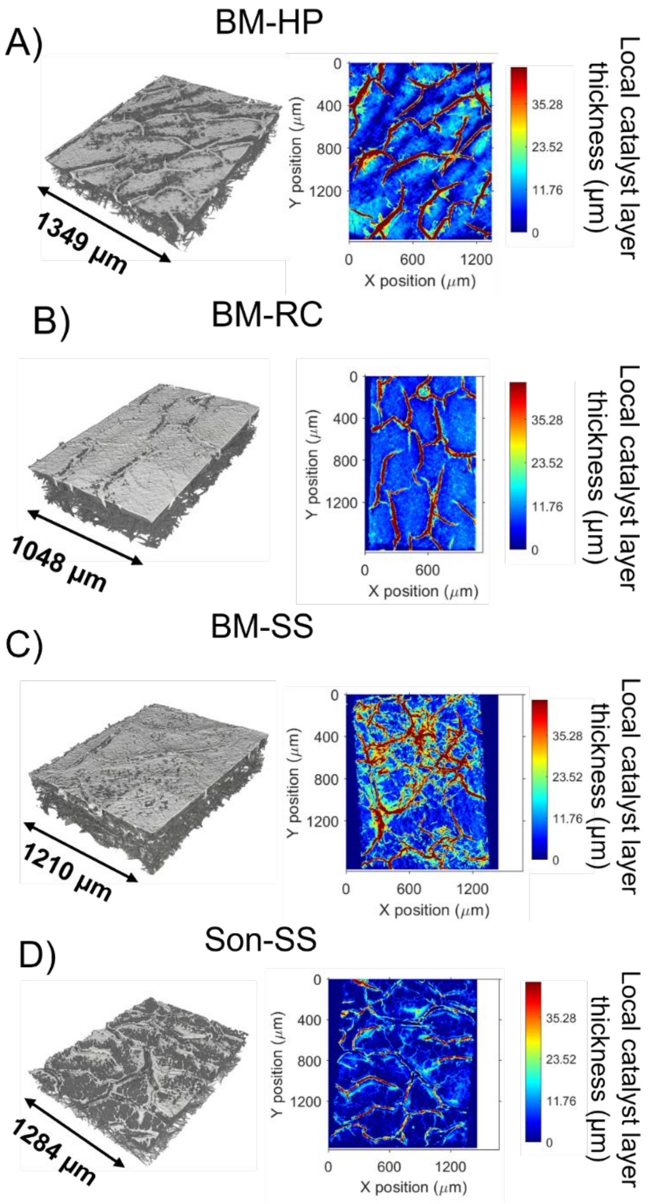


Figure S8. Left: Micro-CT electrode and dimensions, Right: heat map of local catalyst layer thickness of A) BM-HP (ball milled, hand painted), B) BM-RC (ball milled, rod coated), C) BM-SS (ball milled, slow flow rate ultrasonic sprayed), D) Son-SS (sonicated ink, slow flow rate ultrasonic sprayed)

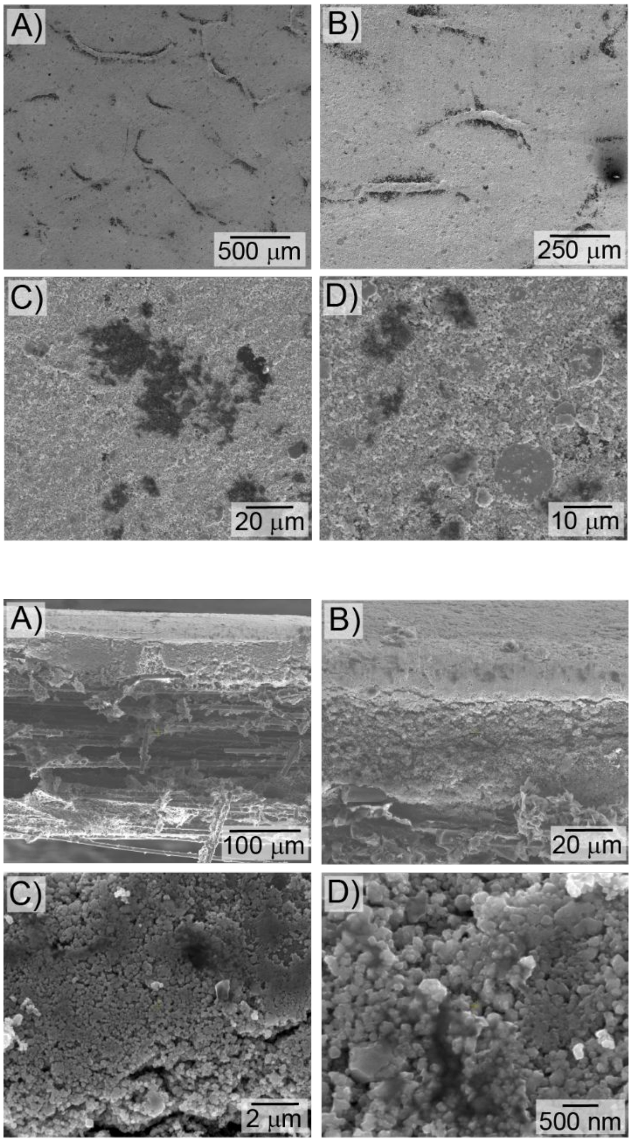


Figure S9: SEM of BM-HP (ball milled, hand painted) electrodes, top: A-D) top down SEM, bottom: A-D) cross sectional SEM

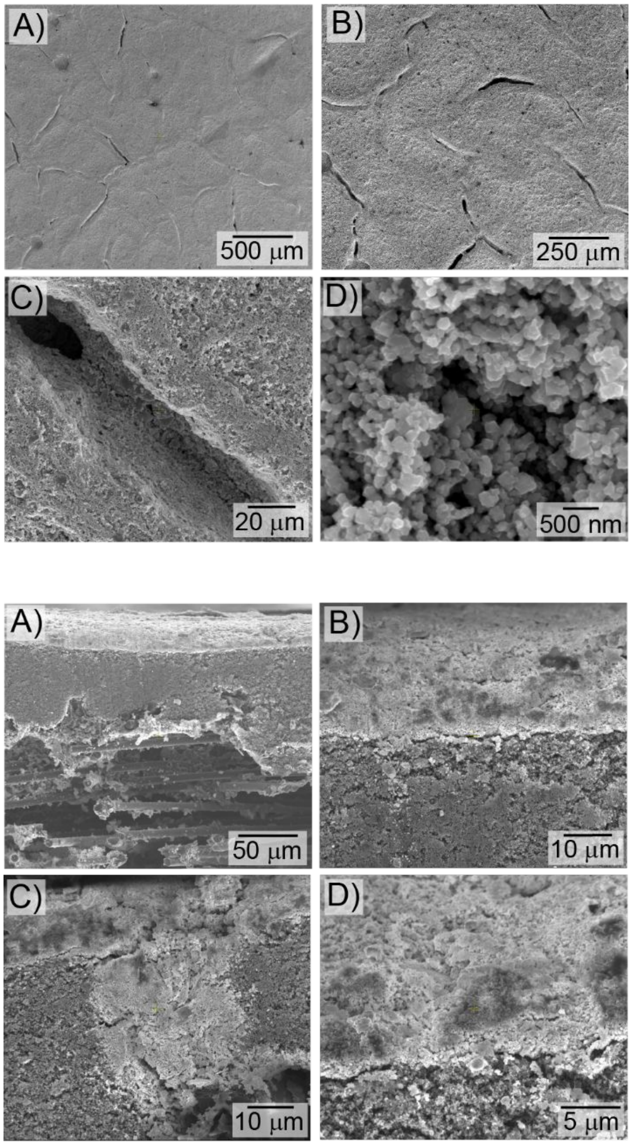


Figure S10: SEM of BM-RC (ball milled, rod coated) electrodes, top: A-D) top down SEM, bottom: A-D) cross sectional SEM

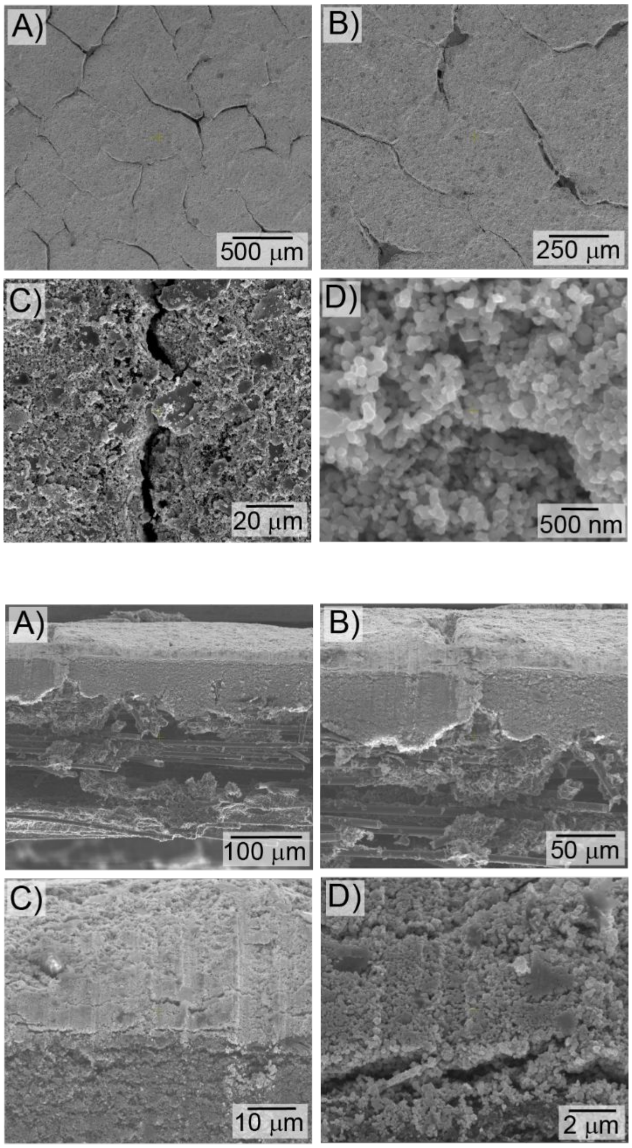


Figure S11: SEM of BM-FS (ball milled, fast sprayed) electrodes, top: A-D) top down SEM, bottom: A-D) cross sectional SEM

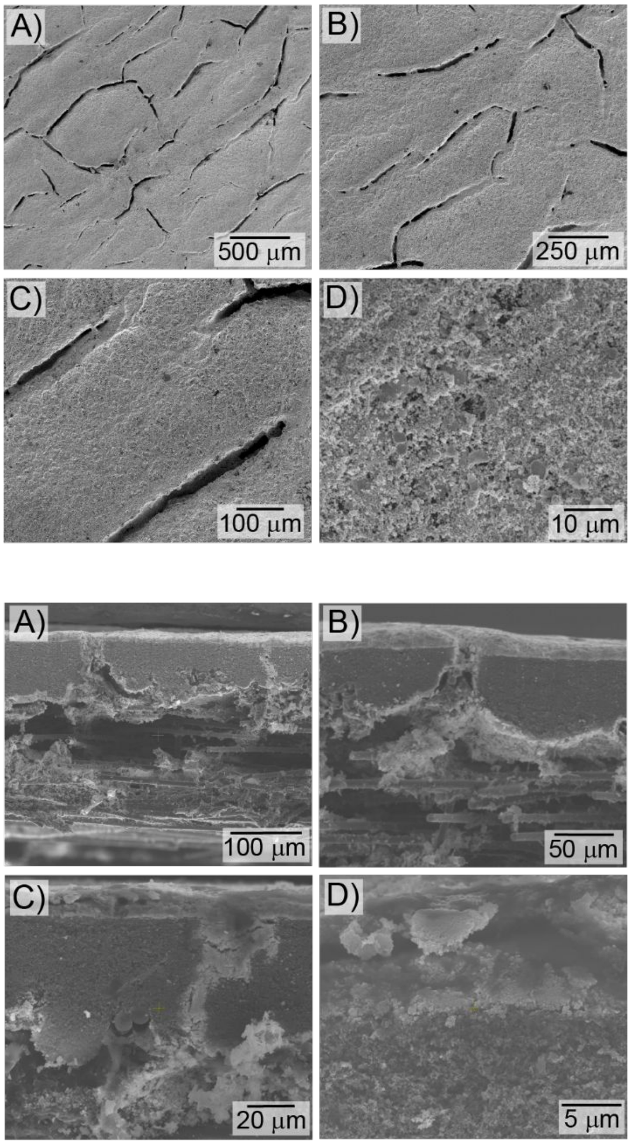


Figure S12: SEM of BM-FS (ball milled, fast sprayed) electrodes, top: A-D) top down SEM, bottom: A-D) cross sectional SEM

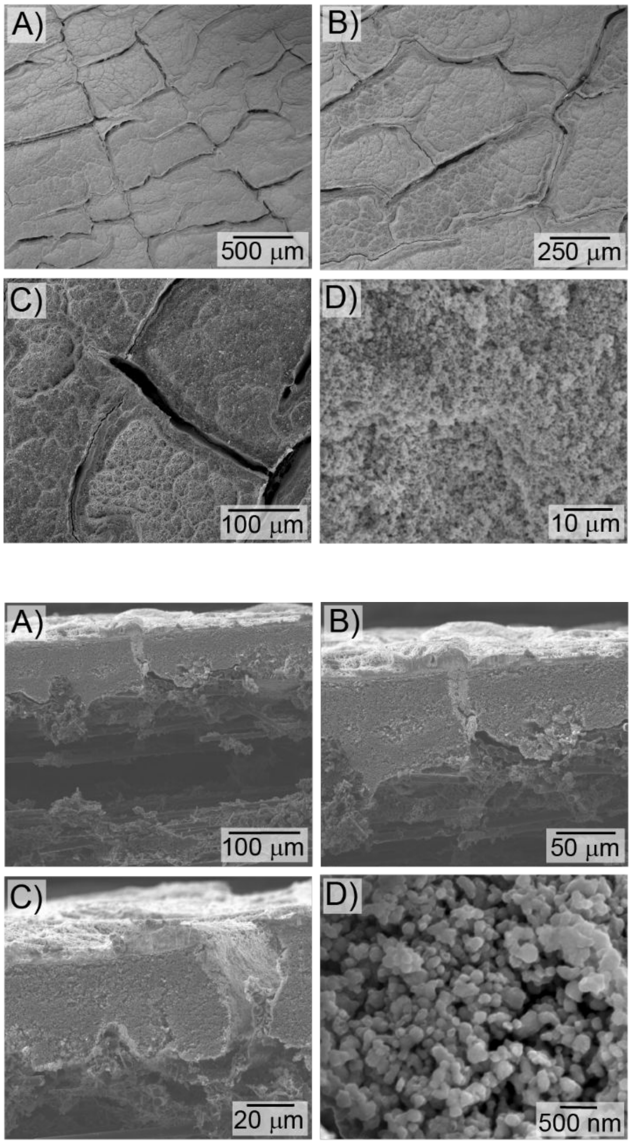


Figure S13: SEM of Son-SS (sonicated, slow flow rate ultrasonic sprayed) electrodes, top: A-D) top down SEM, bottom: A-D) cross sectional SEM

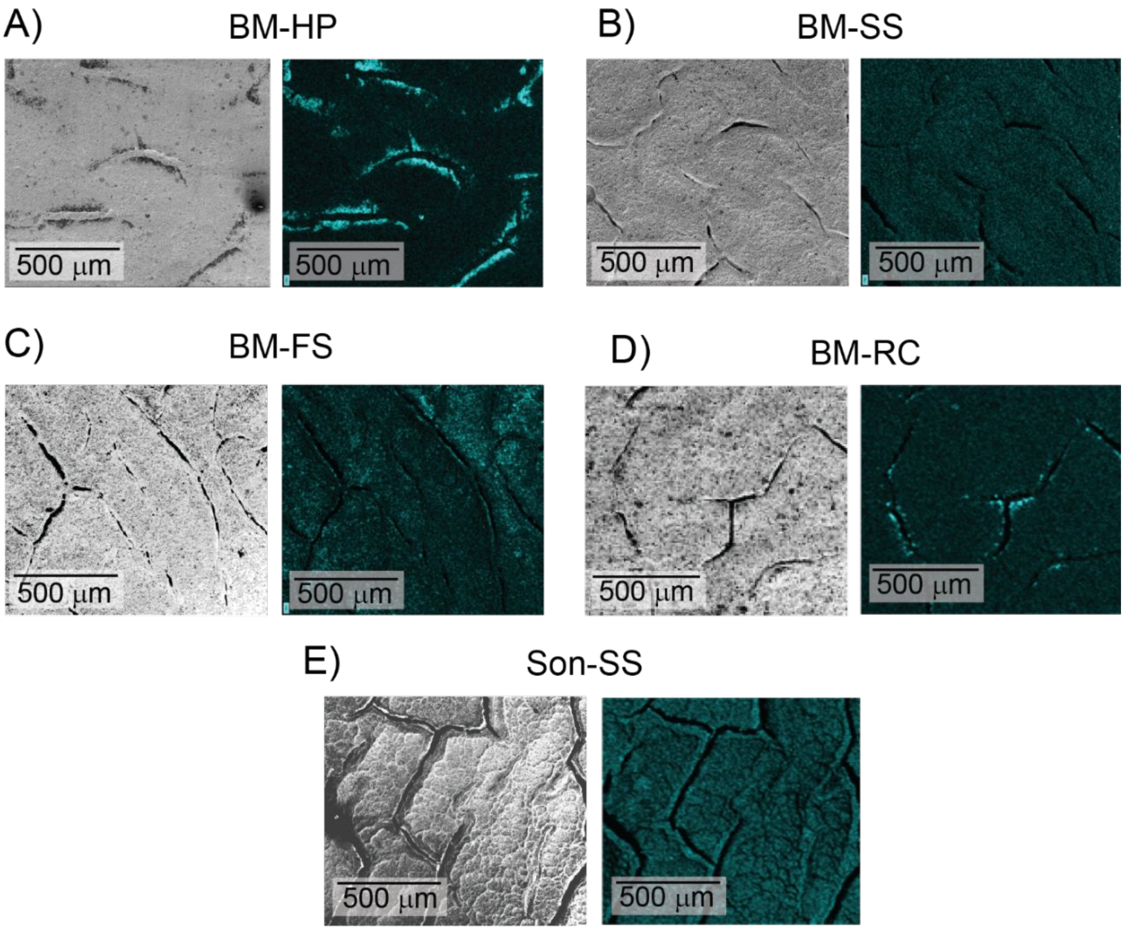


Figure S14. Left: Top-down SEM image, Right: F-EDS map of A) BM-HP (ball milled, hand painted), B) BM-SS (ball milled, slow flow rate ultrasonic sprayed), C) BM-FS (ball milled, fast flow rate ultrasonic sprayed), D) BM-RC (ball milled, rod coated), E) Son-SS (sonicated ink, slow flow rate ultrasonic sprayed)

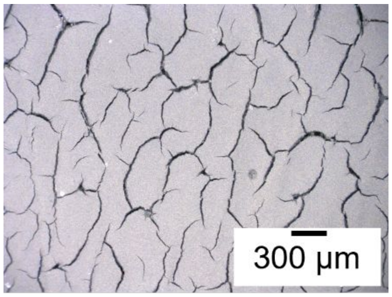


Figure S15. Image of blank Sigracet 39 BB demonstrating cracks inherent in the GDE

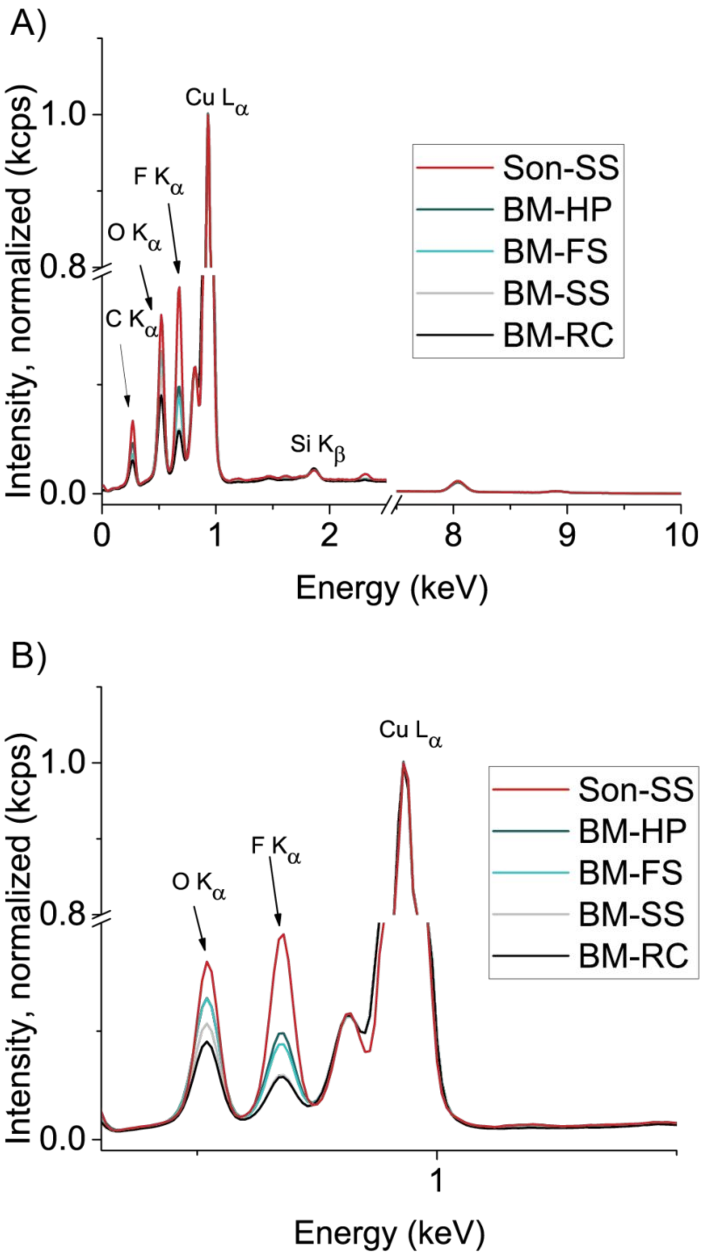


Figure S16: Energy (keV) versus normalized intensity (to Cu) for Son-SS, BM-HP, BM-FS, BM-SS and BM-RC electrodes, A) full spectra, B) inset showing the ratios of F and O to Cu

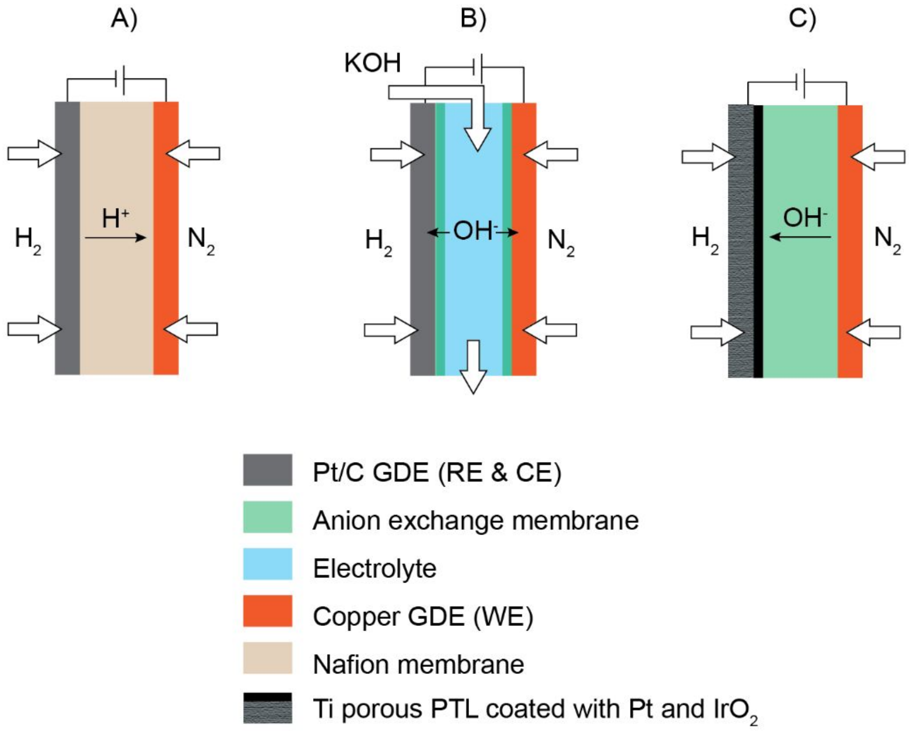


Figure S17: Electrochemical impedance spectroscopy (EIS) configurations to measure Cu GDE properties A) Membrane electrode assembly (MEA) with Nafion® membrane, B) Flowing electrolyte between two anion exchange membranes (AEM) and C) MEA with AEM and Pt/$IrO_2$ PTL (porous transport layer) anode to allow in-situ EIS during cell operation.

Table S2. EIS parameters on capacitance from EIS setup B) from Figure S17

| Sample | Capacitance (µF/cm$^2$) |
|---|---|
| BM SS | 8702 +/- 85 |
| BM RC 6% | 4975 +/- 54 |
| BM RC 0% | 4691 +/- 262 |
| Son SS | 3981 +/- 5 |
| BM FS | 7219 +/- 186 |
| BM HP | 1507 +/-30 |

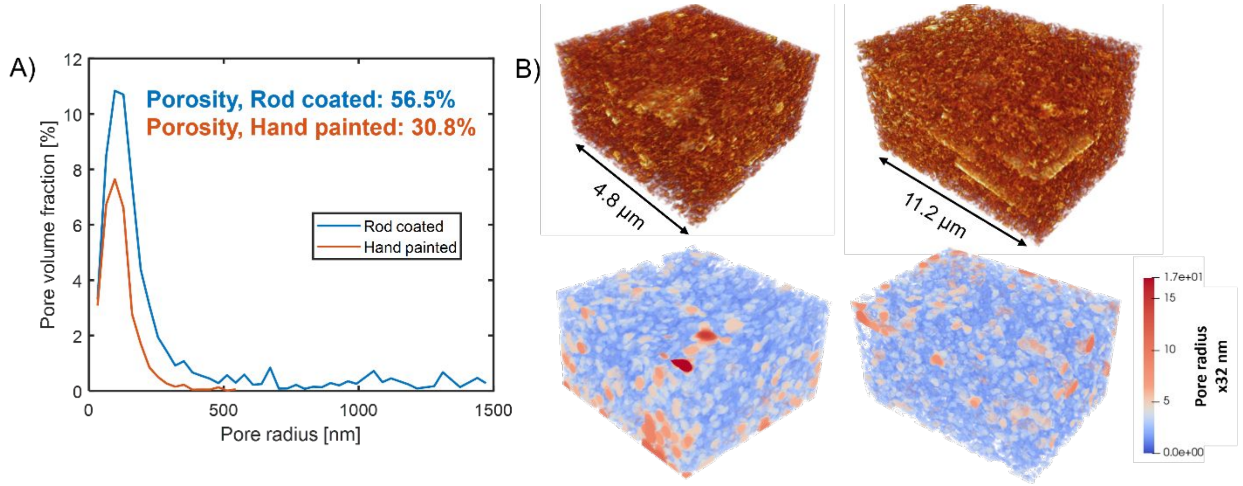


Figure S18. A) Pore volume distribution versus pore radius, B) 3D rendering of solids and pores (left, BM-RC 6%, right, BM-HP 6%)

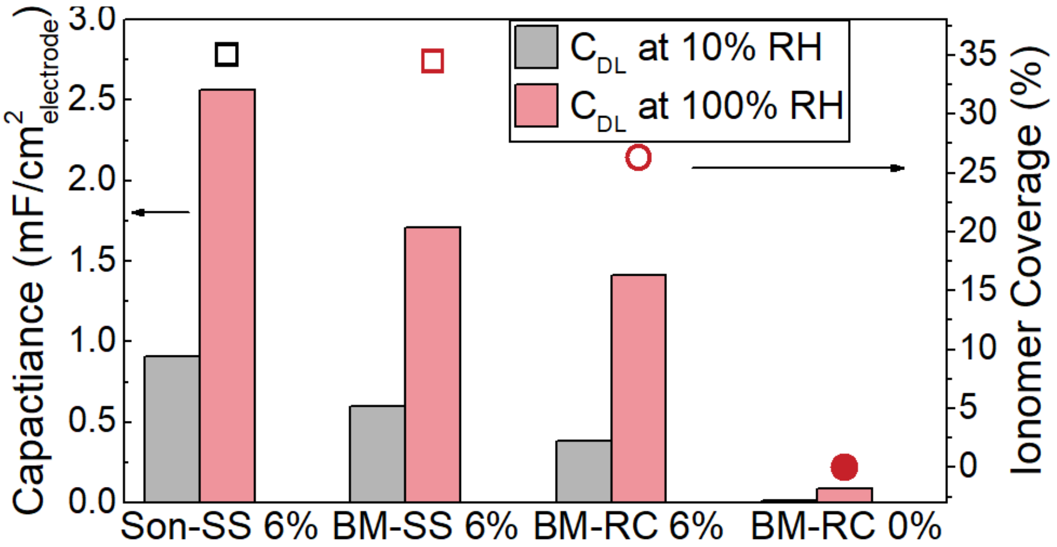


Figure S19. Left y-axis shows capacitance ($mF/cm^2$), right y-axis shows ionomer coverage for Son-SS 6%, BM-SS 6%, BM-RC 6% and BM-RC 0% electrodes.

$$Ionomer\ Coverage = \frac{C_{DL}^{x,10\%} - C_{DL}^{0\%,10\%}}{C_{DL}^{x,100\%}}$$

Equation for ionomer coverage, where $C_{DL}^{x,10\%}$ refers to the double layer capacitance ($C_{DL}$) at 10% RH for the electrode of interest, $C_{DL}^{0\%,10\%}$ refers to the $C_{DL}$ of the BM-RC 0% electrode at 10% RH and $C_{DL}^{x,100\%}$ refers to the $C_{DL}$ at 100% for the electrode of interest.

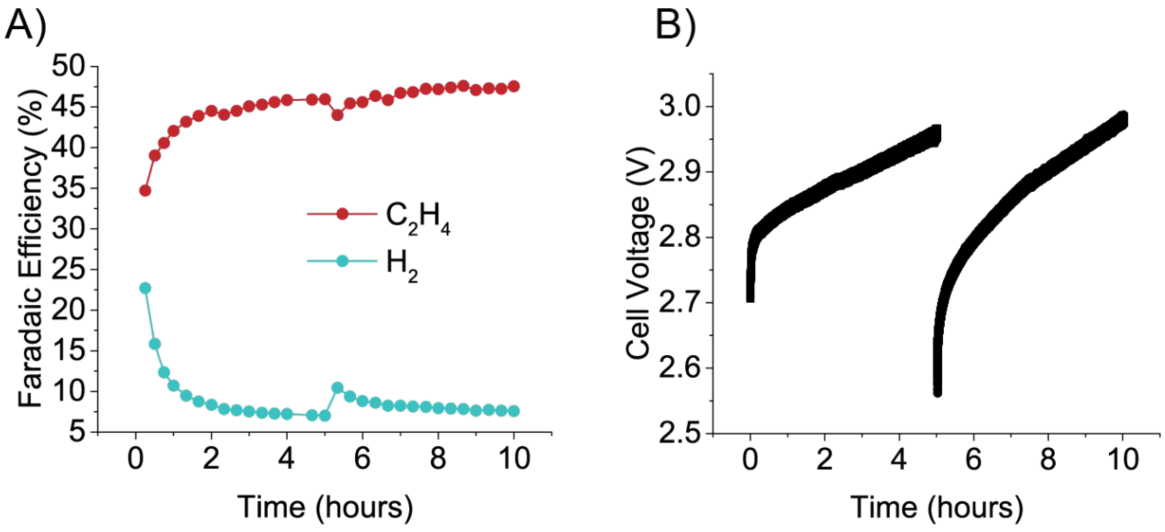


Figure S20: Durability data at 500 mA/cm$^2$ using BM-RC 6% (ball milled, rod coated with 6% Nafion®) electrode, A) ethylene and hydrogen FE%, B) cell voltage data (anolyte was replaced at 5 hours)

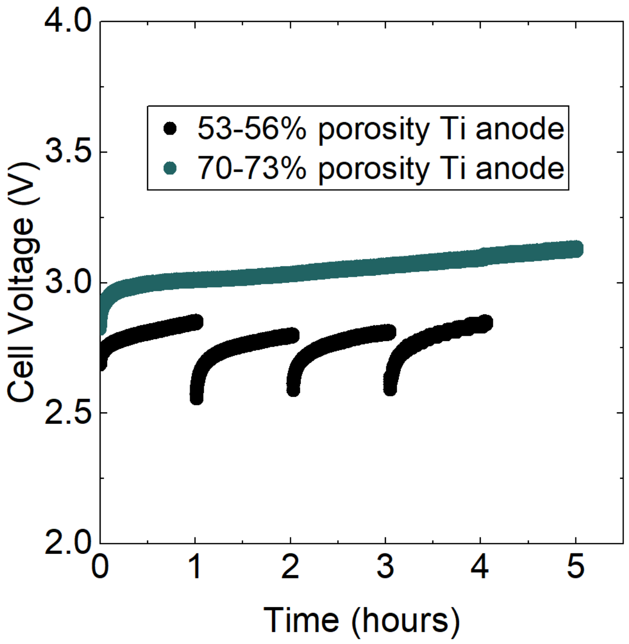


Figure S21. Cell voltage data from Figure 6, for 53-56% and 70-73% porosity anodes from 500 mA/cm$^2$ galvanostatic run. Anode for 53-56% porosity Ti anode was the $IrO_2$ coated Ti mesh anode and the anode for the 70-73% porosity Ti anode was the Pt /$IrO_2$ coated Ti anode. Additionally, EIS were taken every hour and the galvanostatic run was restarted for the 70-73% porosity Ti anode run.

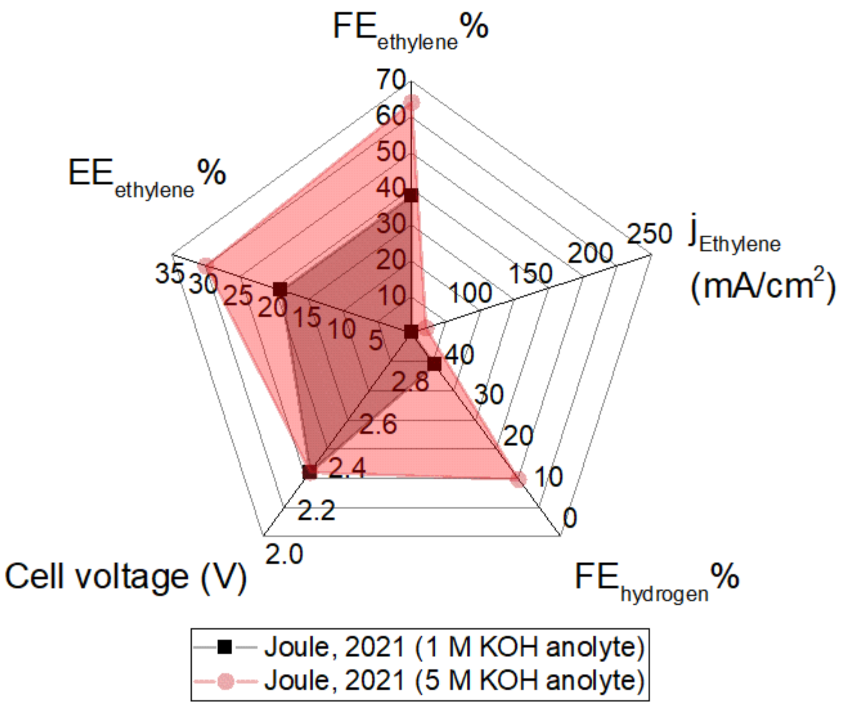


Figure S22: Spider plot comparing ethylene partial current density ($i_{ethylene}$), ethylene Faradaic efficiency ($FE_{ethylene}$%), cell voltage and hydrogen Faradaic efficiency ($FE_{hydrogen}$) with 1 and 5 M KOH anolytes from Ozden et. al. (Joule 2021)[8]. Compared here is the highest $i_{ethylene}$ and metrics associated reported from Ozden et. al.

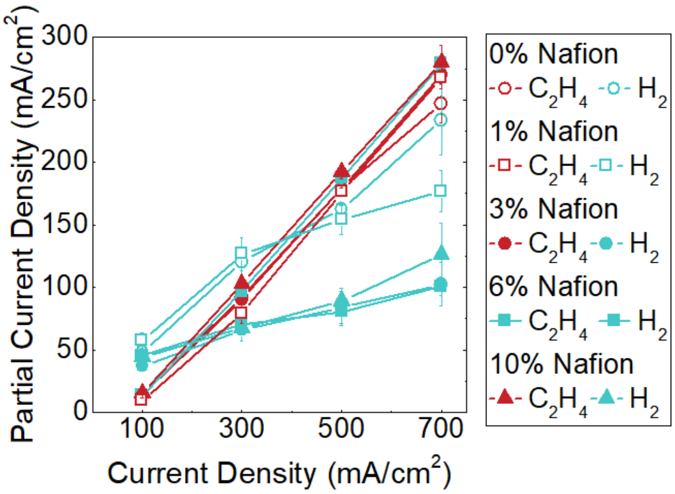


Figure S23: Partial current density (mA/cm$^2$) versus current density for BM-RC 6% (ball-milled, rod-coated with 6% Nafion®) electrodes with varying Nafion® content (0-6%)

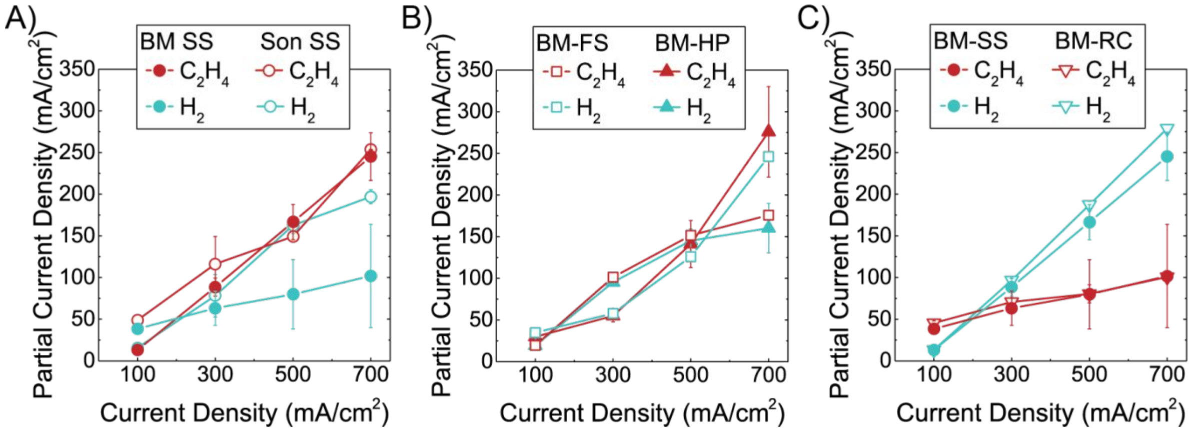


Figure S24: A-C) Partial current density (mA/cm$^2$) versus current density of A) BM-SS (ball milled, slow flow rate ultrasonic sprayed) and Son-SS (sonicated ink, slow flow rate ultrasonic sprayed), B) BM-FS (ball milled, fast flow rate ultrasonic sprayed) and BM-HP (ball milled, hand painted) and C) BM-SS and BM-RC (ball milled, rod coated). Error bars are the standard deviation from 3 independent measurements.

References:


(1) Williams, T. C.; Shaddix, C. R. Contamination of Carbon Monoxide with Metal Carbonyls: Implications for Combustion Research. *Combustion Science and Technology* **2007**, *179* (6), 1225–1230. https://doi.org/10.1080/00102200601057279.

(2) Fiedler, J.; Salmain, M.; Jaouen, G.; Pospíšil, L. Purification of Gaseous CO from Fe(CO)5 Traces Formed in Steel Storage Cylinders. *Inorganic Chemistry Communications* **2001**, *4* (11), 613–616. https://doi.org/10.1016/S1387-7003(01)00287-8.

(3) Saha, P.; Henckel, D.; Intia, F.; Hu, L.; Cleve, T. V.; Neyerlin, K. C. Anolyte Enhances Catalyst Utilization and Ion Transport Inside a CO2 Electrolyzer Cathode. *J. Electrochem. Soc.* **2023**, *170* (1), 014505. https://doi.org/10.1149/1945-7111/acb01d.

(4) Divekar, A. G.; Kuo, M.; Park, A. M.; Motz, A. R.; Page-Belknap, Z. S.; Owczarczyk, Z.; Long, H.; Seifert, S.; Maupin, C. M.; Yandrasits, M. A. The Impact of Alkyl Tri-methyl Ammonium Side Chains on Perfluorinated Ionic Membranes for Electrochemical Applications. *Journal of Polymer Science Part B: Polymer Physics* **2019**, *57* (11), 700–712.

(5) Verdaguer-Casadevall, A.; Li, C. W.; Johansson, T. P.; Scott, S. B.; McKeown, J. T.; Kumar, M.; Stephens, I. E. L.; Kanan, M. W.; Chorkendorff, I. Probing the Active Surface Sites for CO Reduction on Oxide-Derived Copper Electrocatalysts. *J. Am. Chem. Soc.* **2015**, *137* (31), 9808–9811. https://doi.org/10.1021/jacs.5b06227.

(6) Li, Z.; Zhang, T.; Raj, J.; Roy, S.; Wu, J. Revisiting Reaction Kinetics of CO Electroreduction to C2+ Products in a Flow Electrolyzer. *Energy Fuels* **2023**, *37* (11), 7904–7910. https://doi.org/10.1021/acs.energyfuels.3c00736.

(7) Chen, Y.; Vise, A.; Klein, W. E.; Cetinbas, F. C.; Myers, D. J.; Smith, W. A.; Deutsch, T. G.; Neyerlin, K. C. A Robust, Scalable Platform for the Electrochemical Conversion of CO2 to Formate: Identifying Pathways to Higher Energy Efficiencies. *ACS Energy Lett.* **2020**, *5* (6), 1825–1833. https://doi.org/10.1021/acsenergylett.0c00860.

(8) Ozden, A.; Wang, Y.; Li, F.; Luo, M.; Sisler, J.; Thevenon, A.; Rosas-Hernández, A.; Burdyny, T.; Lum, Y.; Yadegari, H.; Agapie, T.; Peters, J. C.; Sargent, E. H.; Sinton, D. Cascade CO2 Electroreduction Enables Efficient Carbonate-Free Production of Ethylene. *Joule* **2021**, *5* (3), 706–719. https://doi.org/10.1016/j.joule.2021.01.007.
